# Single-cell transcriptional pharmacodynamics of trifluridine in a tumor-immune model

**DOI:** 10.1038/s41598-022-16077-7

**Published:** 2022-07-13

**Authors:** Tove Selvin, Erik Fasterius, Malin Jarvius, Mårten Fryknäs, Rolf Larsson, Claes R. Andersson

**Affiliations:** 1 Department of Medical Sciences, Uppsala University, 75185 Uppsala, Sweden; 2 National Bioinformatics Infrastructure Sweden (NBIS), Stockholm University, Stockholm, Sweden; 3 Present Address: Department of Pharmaceutical Biosciences and Science for Life Laboratory, Uppsala University, Box 591, 751 24 Uppsala, Sweden

**Keywords:** Chemotherapy, Cancer microenvironment, Transcriptomics

## Abstract

Understanding the immunological effects of chemotherapy is of great importance, especially now that we have entered an era where ever-increasing pre-clinical and clinical efforts are put into combining chemotherapy and immunotherapy to combat cancer. Single-cell RNA sequencing (scRNA-seq) has proved to be a powerful technique with a broad range of applications, studies evaluating drug effects in co-cultures of tumor and immune cells are however scarce. We treated a co-culture comprised of human colorectal cancer (CRC) cells and peripheral blood mononuclear cells (PBMCs) with the nucleoside analogue trifluridine (FTD) and used scRNA-seq to analyze posttreatment gene expression profiles in thousands of individual cancer and immune cells concurrently. ScRNA-seq recapitulated major mechanisms of action previously described for FTD and provided new insight into possible treatment-induced effects on T-cell mediated antitumor responses.

## Introduction

Progression of colorectal cancer (CRC), as well as many other tumors, involve changes in the tumor microenvironment (TME) toward immunosuppression and loss of immunosurveillance^1^. Additionally, many of the antineoplastic agents used today have adverse effects further suppressing the already challenged immune cells^2,3^. CRC is commonly treated using combined chemotherapies. FDA approved protocols include FOLFOX (5-fluorouracil + oxaliplatin) and FOLFIRI (5-fluorouracil + irinotecan)^4^. For a long time, it was assumed that chemotherapy had only immunosuppressive adverse effects on the immune system; however, this viewpoint has shifted as certain chemotherapeutics have been shown to enhance tumor-specific immune responses^5,6^. Preclinical data suggest that FOLFOX reduces immunosuppression while FOLFIR augments immunosuppression^7^. Furthermore, clinical data indicate that FOLFOX treatment results in higher overall survival rates^8^, potentially linked to its favorable immunoregulatory effect^6^. Taken together, it has become evident that treatment outcome is dictated by the combined effect on both cancer- and immune cells^3,5^, making it crucial to improve our knowledge regarding the interplay between chemotherapeutic agents and the immune system.

The concept of immunogenic cell death (ICD) induced by antineoplastic agents has gained increasing interest^6^. ICD is a form of regulated cell death (RCD) capable of activating an adaptive immune response by provoking the release of damage-associated molecular patterns (DAMPs)^9^. Today, oxaliplatin is an acknowledged ICD inducer^10^ and this property has been demonstrated both in preclinical^11^ and clinical settings^12^. The nucleoside analogue trifluridine (FTD) is the active component of TAS-102; an antitumor drug first approved for treatment of metastatic colorectal cancer (mCRC) in Japan 2015^13,14^. Thus far, not much is known about the immunological effects of FTD, a recent study by Limagne et al. did however demonstrate that FTD is capable of inducing ICD in various human CRC cell lines in vitro^15^.

Gene expression profiles are a highly informative phenotypic measure of treatment response^16,17^. Until recently each cell type of interest had to be sequenced separately and with bulk expression profiles as a read-out. Today, sequencing with single-cell resolution enables thorough evaluation of heterogeneous responses across multiple cell types and subsets of cell populations simultaneously^18–20^. However, there is a lack of studies exploring drug effects in co-cultures of tumor and immune cells using this technique. To address this and to learn more about the impact of FTD on both cancer- and immune cells, we co-cultured the human CRC cell line HCT116 with peripheral blood mononuclear cells (PBMCs) and used Single-Cell 3′ RNA sequencing (scRNA-seq) to analyze posttreatment gene expression profiles in thousands of individual cancer and immune cells concurrently. ScRNA-seq recapitulated major mechanisms of action previously described for FTD and provided new insight into possible treatment-induced effects on T-cell mediated antitumor responses.

## Results

### Trifluridine induces the release of ICD markers and secretion of pro-inflammatory cytokines in vitro

**Figure 1:**
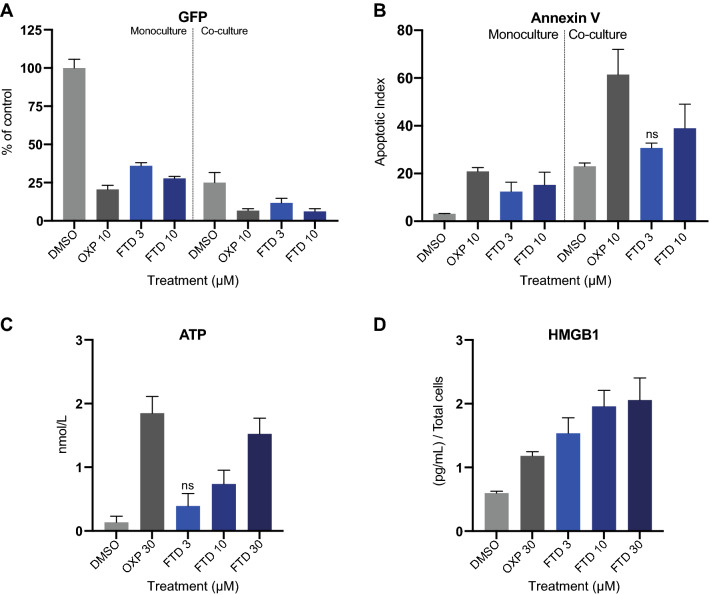
Trifluridine decreases viability and increases release of ICD markers in vitro. (**A**) Viability indirectly measured by image based GFP quantification (n = 9) and (**B**) Apoptotic Index determined by Annexin V (number of Annexin V positive cells/number of GFP expressing cells) (n = 6) in HCT116-GFP monoculture and co-culture with human PBMCs, treated with DMSO vehicle (0.1%), oxaliplatin (OXP) (10 μM), or trifluridine (FTD) (3, 10 μM) for 72 h. (**C**) ATP measured by CellTiter Glo in supernatants of HCT116-GFP monoculture treated for 48 h (n = 6) and (**D**) HMGB1 measured by ELISA in supernatants of HCT116-GFP monoculture treated for 72 h. Data normalized against total number of cells per sample. Results are shown as mean ± SD from two (**B**–**D**) or three (**A**) independent experiments. Compared to DMSO vehicle, P ≤ 0.05 (One-way Anova with Dunnett’s multiple comparison test) for all treatment data sets except the ones marked ns, not significant.

Treatment response and ICD induction of FTD and positive control oxaliplatin (OXP) were evaluated in the CRC cell line HCT116-GFP, cultured as monoculture or co-cultured with human PBMCs stimulated with anti-CD3 and IL-2. Treatment with FTD or OXP for 72 h effectively decreased viability (indirectly measured as GFP signal) (Fig. 1A) and increased apoptosis (Annexin V) (Fig. 1B) in both mono- and co-culture. FTD has previously been shown to induce ICD in various human CRC cell lines in vitro^15^. Therefore, we measured two gold standard in vitro ICD markers, secretion of ATP and release of HMGB1^21^, and found that FTD significantly and dose-dependently increased both extracellular ATP (Fig. 1C) and HMGB1 (Fig. 1D). These data suggest ICD induction also in our model system. As HMGB1 is overexpressed and secreted in colon cancers^22^, HMGB1 levels were normalized against the total number of cells in each sample (unnormalized data and cell count, Supplementary Fig. 1).

**Figure 2:**
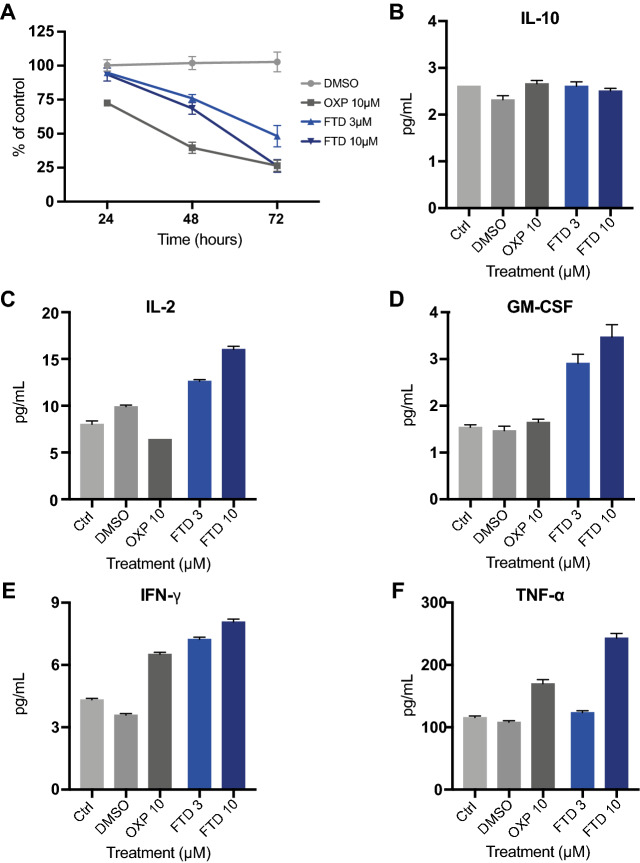
Trifluridine increases secretion of pro-inflammatory cytokines. (**A**) Viability measured over time by GFP expression in HCT116-GFP co-culture with human PBMCs, treated with DMSO vehicle, OXP, or FTD (n = 9). Cytokines (**B**) IL-10, (**C**) IL-2, (**D**) GM-CSF, (**E**) IFN-γ, and (**F**) TNF-α measured in supernatants of HCT116-GFP co-cultured with human PBMCs and treated for 48 h using a Luminex MAGPIX system (technical triplicates).

When evaluating treatment responses in co-culture over time, OXP had a greater effect than FTD at earlier time-points, whereas after 72 h exposure the result was equivalent (Fig. 2A). Many chemotherapeutic drugs have immunosuppressive adverse effects and the ability to increase tumor immunogenicity by inducing ICD does not exclude other off-target effects on the immune system^3,23,24^. To further evaluate the impact of FTD in the co-culture system, a Luminex MAGPIX system was used to measure cytokine release 48 h post-treatment. Neither FTD nor OXP affected the secretion of anti-inflammatory cytokine IL-10 (Fig. 2B). Furthermore, FTD dose-dependently increased the secretion of pro-inflammatory cytokines interleukin (IL)-2, granulocyte–macrophage colony-stimulating factor (GM-CSF), interferon-gamma (IFN-γ), and tumor necrosis factor alpha (TNF-α) (Fig. 2C–F).

### ScRNA-seq captures major mechanisms of action of trifluridine

**Figure 3:**
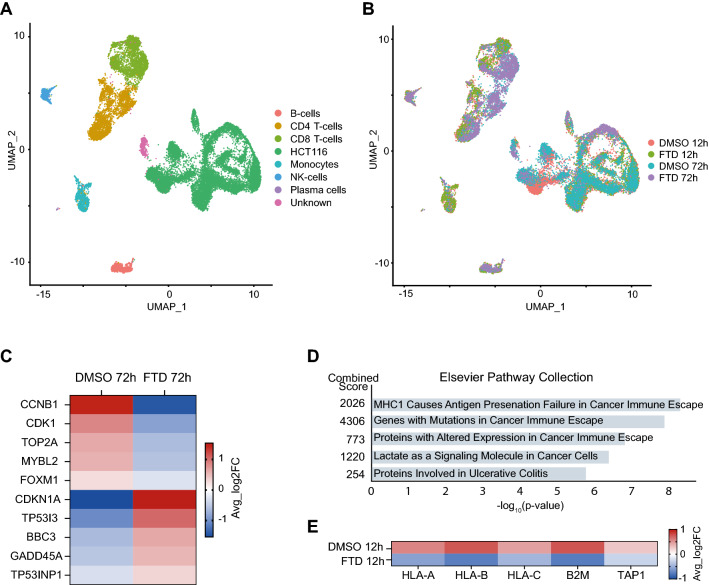
scRNA-seq captures major mechanisms of action of trifluridine. UMAP projection of all cells colored by (**A**) cell type and (**B**) sample identity. (**C**) Heatmap showing average Log2 fold change expression of genes of interest in HCT116 treated with DMSO vehicle (0.1%) or FTD (3 μM) for 72 h. (**D**) Top 5 enriched pathways obtained from pathway enrichment analysis using the “Elsevier Pathway Collection” database. The analysis was performed with 52 DEGs (Adjusted P-value < 0.001) identified for HCT116 treated with FTD for 12 h compared to DMSO vehicle. (**E**) Heatmap showing average Log2 fold change expression of genes of interest in HCT116 treated with FTD for 12 h compared to DMSO vehicle.

The field of single-cell transcriptomics is rapidly growing as scRNA-seq has proved to be a powerful technique with a broad range of applications^25^, studies evaluating drug effects in co-cultures of tumor and immune cells are however scarce. In the present study, Chromium Next GEM scRNA-seq of HCT116 co-cultured with PBMCs and treated with DMSO vehicle (0.1%) or FTD (3 μM) for 12 h or 72 h was performed. After quality control and filtering, the transcriptomes of 12,824 cancer cells and 8,432 immune cells were obtained and visualized using Uniform Manifold Approximation and Projection (UMAP). Based on cell-type specific gene expression markers, cell clusters were manually annotated as B cells, CD4^+^ T cells, CD8^+^ T cells, HCT116, monocytes, and NK cells (Fig. 3A).

FTD has previously been shown to inhibit *MYC*^26^, an oncogene known to promote cell cycle progression^27^, along with members of the *E2F*-family and downstream *E2F* targets such as Forkhead Box M1 (FOXM1) and B-Myb (MYBL2)^26^, both of which are proliferation-associated transcription factors commonly overexpressed in various cancers^28,29^. Furthermore, FTD has been shown to activate the p53 pathway^30,31^, a pathway whose activation upon DNA damage can lead either to induction of p21 (*CDKN1A*) and subsequent cell cycle arrest or to apoptosis mediated by p53 upregulated modulator of apoptosis (*BBC3*) and TP53-inducible gene 3 (*TP53I3*)^31^. All of the above-mentioned mechanisms of action were captured by scRNA-seq. Differential gene expression analysis of CRC cells (HCT116) treated with DMSO vehicle or FTD for 72 h (Fig. 3B) revealed 1150 differentially expressed genes (DEGs) (Adjusted P-value < 0.05) (Supplementary data 1). Performing pathway enrichment analysis, using the MSigDB Hallmark 2020 database, on the complete set of DEGs identified E2F Targets, Myc Targets, G2-M Checkpoint, p53 Pathway, and apoptosis among the top 10 enriched terms (Supplementary data 2). Upon a closer look at the expression of key genes involved in these pathways, significantly lower expression of Cyclin B1 (*CCNB1*), cyclin-dependent kinase 1 (*CDK1*), topoisomerase II (*TOP2A*), *MYBL2*, and *FOXM1*, whose inhibition is associated with decreased proliferation and G2 arrest^28,29,32^, was observed in cells treated with FTD compared to cells treated with DMSO vehicle. Concordantly, cells treated with FTD also had higher expression of *TP53I3*, *CDKN1A*, *BBC3* and *GADD45A* (Fig. 3C).

### ScRNA-seq suggests that trifluridine treatment dampens T cell-mediated antitumor responses

Differential gene expression analysis of CRC cells treated with DMSO vehicle or FTD for 12 h (Fig. 3B) revealed 52 DEGs (Supplementary data 1). Pathway enrichment analysis identified pathways involved in cancer immune escape as the top three enriched terms (Fig. 3D). Cells treated with FTD had lower expression of all three classical human leukocyte antigen class I (HLA-I) molecules (HLA-A, HLA-B, and HLA-C) (Fig. 3E). HLA-I plays a crucial role in presenting processed tumor antigens to T cells and tumor cell loss of HLA-I is well established as a mechanism for escaping T cell-mediated antitumor responses^33,34^. Furthermore, FTD-treated cells had lower expression of *B2M*, a gene encoding the β2M protein responsible for stabilizing the HLA-I complex^34^, and *TAP1*, a component of the antigen-presenting machinery (APM) whose down-regulation is associated with tumor immune escape and worse prognosis in CRC patients^35^. These results indicate that although FTD treatment effectively decreases the viability of tumor cells in vitro, tumor-promoting effects might also be exerted by facilitating tumor immune escape.

Differential gene expression analysis of CD4^+^ and CD8^+^ T cells revealed 35 and 37 DEGs, respectively, after 72 h treatment with FTD compared to DMSO vehicle (Supplementary data 1). For CD4^+^ T cells, the gene with the highest fold change increase in FTD-treated cells was B-cell translocation gene 1 (*BTG1*), also known as BTG anti-proliferation factor 1, a negative regulator of the cell cycle^36^. BTG1 has also been shown to play an essential role in suppressing the activation of quiescent T cells^37^. In CD8^+^ T cells, the highest fold change increase in treated cells was observed for *SLA*, a gene encoding the Src-like adaptor protein which has been described as a negative regulator of T cell receptor (TCR) signaling^38^. Increased mRNA levels of BTG1 and SLA in T cells, together with decreased expression of HLA- I molecules in tumor cells, suggest that FTD treatment might dampen T cell-mediated antitumor responses both by facilitating tumor immune escape and by reducing proliferation capacity and TCR signaling in T cells. Additionally, in both CD4^+^ and CD8^+^ T cells, *MT2A* was found among DEGs with decreased expression in FTD-treated cells compared to DMSO (Supplementary data 1). Metallothioneins (MTs), including metallothionein 2A encoded by the *MT2A* gene, is a family of metal-binding proteins that has, in recent years, emerged as important modulators of both innate and adaptive immunity^39^. For B-cells, monocytes, and NK cells, treatment with FTD did not induce any pro- or anti-inflammatory effects that were apparent when analyzing mRNA expression.

### Trajectory analysis recapitulates p53-mediated cell cycle arrest

**Figure 4:**
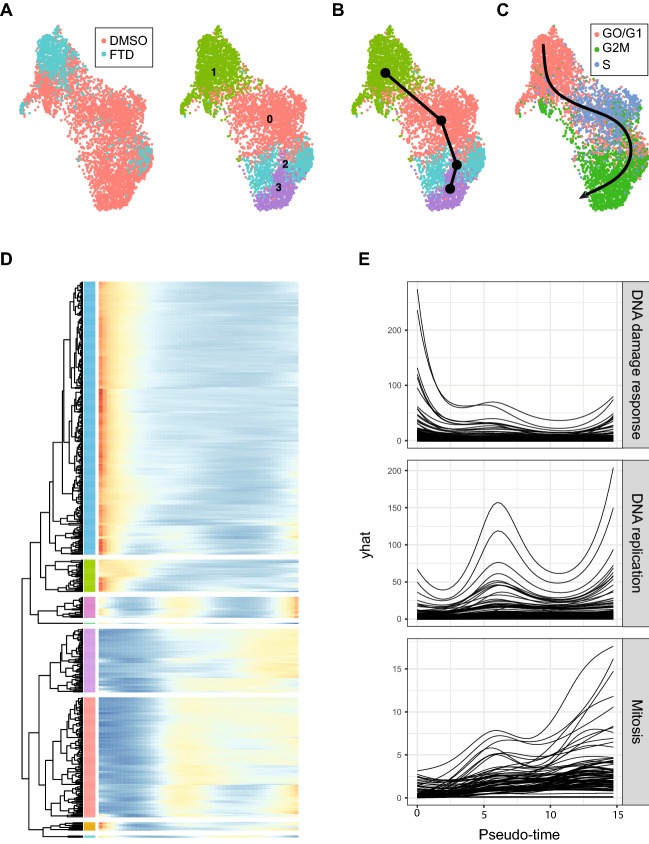
Trajectory analysis recapitulates p53-mediated cell cycle arrest. (**A**) UMAP projection of HCT116 cells at 72 h, colored by sample identity (left) and clustering (right), showing enrichment of treated cells in a subset of clusters. (**B**) Trajectory inference on this clustering identified a single lineage. (**C**) Trajectory curve overlayed on inferred cell cycle phase suggests an association between pseudo-time and cell cycle dependency. Direction of trajectory indicated by arrowhead. (**D**) Heatmap of pseudo-time vs gene expression for genes that discriminate between FTD and DMSO treated cells where the genes have been clustered. Each row corresponds to a gene and each column to a pseudo-time point with the starting point at the left. (**E**) Fitted pseudo-time—gene expression curves for the three largest sub-clusters.

Experience from bulk transcriptomic data has demonstrated that studying the transcriptional response in response to treatment can provide insight into the mode of action of compounds^17^. Here we hypothesized that it is possible to construct single-cell trajectories^40^ where the inferred pseudo-time corresponds to varying degrees of progression of response to treatment. A preliminary analysis indicated relatively few cells with a distinct transcriptional signature induced by FTD treatment at 12 h (see Supplementary Fig. 2), hence we focused on HCT116 at the 72 h time-point where the difference was more pronounced. Unsupervised clustering of cells from control and treatment produced a cluster highly enriched for treated HCT116-cells (Fig. 4a). Trajectory inference on this clustering identified a single lineage (Fig. 4b). To clarify the relationship between the trajectory and cell cycle phase, the trajectory curve was overlayed on inferred cell cycle phase (Fig. 4c). It appears as if the clustering is mainly driven by the cell cycle state of the cells which is then recapitulated in the trajectories with pseudo-time roughly corresponding to cell cycle progression. Furthermore, few treated cells were inferred to be in an actively replicating state (G2/M or S) and in addition, treated cells were distinct within the population of cells inferred as G1 (i.e., not G2/M or S) and extreme with respect to the total HCT116 cell population at 72 h.

To further investigate the dynamics with respect to pseudo-time of genes related to treatment, we identified a set of 500 genes that discriminate control and treated cells using a non-linear classifier and fitted pseudo-time-gene expression curves for the gene set. The fitted curves were then clustered (Fig. 4d), identifying gene clusters that peak at the beginning, middle and end with respect to pseudo-time. Gene set enrichment analysis was performed for each of the subclusters (Supplementary data 3). The analysis identified genes associated with mitosis and DNA synthesis in line with the orientation and direction of the trajectory (Fig. 4e). Thus, many of the genes that discriminate treated cells correspond to normal cell cycle progression. This is likely due to the induction of cell cycle arrest upon FTD treatment. For genes that peak where treated cells are enriched (which includes *CDKN1A*) there was an association with DNA damage response. Thus, single-cell pseudo-time analysis appears to recapitulate an FTD-induced cycle arrest with concomitant induction of p21 in the HCT116 cells.

## Discussion

Studying immunological effects of chemotherapy is of great importance, especially now that we have entered an era where ever-increasing pre-clinical and clinical efforts are put into combining chemotherapy and immunotherapy to combat cancer. FTD has limited bioavailability and is rapidly degraded via thymidine phosphorylase; therefore, in the clinic FTD is given as an oral combination tablet also containing a thymidine phosphorylase inhibitor, tipiracil hydrochloride^41^. This improves the bioavailability considerably and plasma concentrations exceeding 15 µM are clinically achievable at standard doses^42^. At clinically relevant concentrations, we observed an additive antitumor activity of FTD and anti-CD3/IL-2 stimulated PBMC in co-culture (Fig. 1A,B). In agreement with Limagne et al.^15^, we also observed an increased secretion of ICD markers following FTD treatment and we demonstrated a dose-dependent increase of pro-inflammatory cytokines in co-culture (Fig. 2C–F). These results suggest a favorable immunological effect of FTD treatment.

Since RNA-sequencing was developed over a decade ago it has revolutionized our molecular understanding of cells. The refinement from bulk sequencing to single-cell resolution has further fueled discoveries and innovations in medicine over the last few years^43^. In the present study we explored the feasibility of utilizing scRNA-seq to simultaneously study transcriptional drug responses in cancer- and immune cells. The observed FTD-induced effects on CRC cell gene expression in our study were concordant with the literature. FTD has for example been shown to exert at least part of its antitumor effect by activation of the p53 pathway, resulting in p21 induction and cell cycle arrest^31^, a mechanism recapitulated by scRNA-seq (Fig. 3C). ScRNA-seq also demonstrated that CRC cells treated with FTD have a lower expression of HLA-I molecules, B2M, and TAP1 (Fig. 3E), all involved in antigen presentation and commonly downregulated in cancers as a mechanism of immune escape^33,44–46^. Furthermore, higher expression of genes encoding negative regulators of the cell cycle and TCR signaling was observed in T cells treated with FTD, further supporting that FTD could dampen T cell-mediated antitumor responses. These results suggest that using single-cell transcriptomics to study immunological effects of chemotherapy is a feasible approach. However, although our model system incorporates both cancer and immune cells, it has shortcomings in regard to reflecting the TME in colorectal cancer patients. Moving forward, applying this approach on a more complex 3-dimensional (3D) model system, preferably one that also incorporates stromal cells and extracellular matrix (ECM) components, could increase the clinical translatability of findings.

A popular mode of analysis in single-cell transcriptomics is trajectory analysis; a methodology originally devised to identify cell lineages^40^. Here we investigated the hypothesis that it is possible to construct single-cell trajectories where the inferred pseudo-time corresponds to varying degrees of progression of response to treatment. For cytotoxic compounds specifically, bulk gene expression data at early time-points can be used to identify the mechanism of action^17^ whereas later time-points are dominated by cell death. The basic idea here was that single cell gene expression data at a single time-point would contain a snapshot of cells in various degrees of progression towards cell death and thus could facilitate the identification of early, intermediate and late gene expression changes in a single experiment. However, here we were not able to further characterize treatment-induced gene expression changes as early, intermediate or late effects using trajectory analysis. This could be due to the experimental design. The 12 h time-point was presumably too early given that FTD has a cell cycle-dependent mechanism, and at 72 h most HCT116 cells displayed characteristics of cell cycle arrest. Thus, an intermediate time-point might have been more informative, a time-point where a large subset but not all cells would have cycled through cell cycle checkpoints.

Taken together, scRNA-seq of co-cultured cancer- and immune cells treated with FTD did recapitulate mechanisms of action previously described for FTD and provide new insight into possible treatment-induced effects on T-cell mediated anti-tumor responses. However, we believe that optimization of the experimental design could further increase the information output. In conclusion, although FTD appears to improve the overall antitumor activity of anti-CD3/IL-2 stimulated PBMCs in co-culture, our results demonstrate that immunosuppressive effects may also be exerted. How these opposing effects translate to the in vivo situation remains to be elucidated.

## Methods

### Cell culture

The human CRC cell line HCT116 was purchased from ATCC (Manassas, VA, USA) and HCT116-GFP, the same cell line constitutively expressing green fluorescent protein, was obtained from AntiCancer Inc. (San Diego, CA, USA). Cells were cultured in McCoy's 5A Medium, supplemented with 10% heat-inactivated fetal bovine serum (FBS), 2 mM l-glutamine, and Penicillin (100 U/mL)/Streptomycin (100 μg/mL) (all from Sigma, St Louis, MO, USA) at 37 °C in 5% CO_2_. PBMCs from anonymous, healthy donors were isolated by Histopaque-1077 (Sigma) density gradient centrifugation and stored in FBS supplemented with 10% DMSO in − 150 °C until used.

### Materials

Trifluridine (FTD) and Oxaliplatin (OXP) were purchased from Sigma. FTD was dissolved in DMSO and kept as 10 mM stock solution. OXP was purchased as 12.6 mM stock solution in sterile water. The compounds were diluted with culture medium to desired concentrations. Anti-human CD3 was purchased from Affymetrix (Santa Clara, CA, USA) and IL2 from Peprotech (Rocky Hill, NJ, USA).

### Viability and apoptosis measurements

Monocultures were established by seeding HCT116-GFP in 96-well Nunc plates (5000 cells/100μL/well). To establish co-cultures, HCT116-GFP were seeded at the same density as in monoculture and precultured for 24 h before PBMCs were added. PBMCs were thawed and resuspended in McCoys’s 5A Medium supplemented with anti-CD3 (final concentration 100 ng/mL) and IL2 (final concentration 10 ng/mL). PBMCs were then seeded together with precultured cancer cells at a 1:8 ratio (cancer cells: PBMCs). Mono- and co-cultures were treated with DMSO vehicle (0.1%), OXP (3 μM, 10 μM), or FTD (3 μM, 10 μM) and placed in the Live-Cell Analysis System IncuCyte S3 from Essen Bioscience (Ann Arbor, MI, USA). Viability was monitored by measuring GFP expression. Apoptosis was measured by detection of phosphatidylserine exposed on the extracellular surface using Annexin V Red Dye (Essen Bioscience).

### Detection of ICD-markers

HCT116-GFP were seeded in 96-well Nunc plates (15,000 cells/100μL/well for ATP measurements and 5000 cells/100μL/well for HMGB1 measurements). Cells were treated with DMSO vehicle (0.1%, 0.3%), OXP (3 μM, 10 μM, 30 μM), or FTD (3 μM, 10 μM, 30 μM) and supernatants were collected after 48 h and 72 h for subsequent ATP and HMGB1 measurements. Extracellular ATP levels were measured using CellTiter-Glo (CTG) (Promega, Madison, WI, USA). Equal volumes of supernatant and CTG solution were added to a 96-well plate along with ATP standard (BioThema, SWE) and placed on a shaker for 2 min before luminescence was measured using a FLUOstar Omega (BMG Labtech GmbH, Offenburg, DEU). The HMGB1 level in cell culture supernatants was measured using ELISA (Nordic BioSite, SWE). Cell culture supernatants were pre-diluted in PBS (1:5) and further diluted in Dilution Buffer (final dilution 1:50) in a 96-well plate pre-coated with capture antibody. ELISA was performed according to manufacturer’s instructions and absorbance was measured at 450 nm using a FLUOstar Omega. HMGB1 levels were normalized against the total number of cells in each well, determined by cell count using the Live-Cell Analysis System IncuCyte S3 prior to collecting supernatants.

### Measurement of cytokines

HCT116-GFP (5000 cells/100μL/well) were seeded in 96-well Nunc plates and co-cultured with unstimulated PBMC (i.e., no addition of anti-CD3 or IL-2). Co-cultures were treated with DMSO vehicle (0.1%), OXP (10 μM), or FTD (3 μM, 10 μM) for 48 h before supernatants were collected. Cytokine levels in the supernatants were measured using a Bio-Plex Pro Human Cytokine 8-plex assay (Bio-Rad, USA) according to the manufacturer’s instructions. Briefly, the cytokines of interest are bound to magnetic beads via antibodies and subsequently detected using biotinylated antibodies with a fluorescent reporter. Fluorescence was measured using a Luminex MAGPIX system (Bio-Rad, USA) and concentrations were calculated using standard curves.

### Chromium Next GEM single Cell 3′ RNA-sequencing

HCT116 were seeded in 6-well Nunc plates (50,000 cells/3 mL/well) and precultured for 24 h before PBMCs were added at a 1:8 ratio. Co-cultures were treated with DMSO vehicle (0.1%) or FTD (3 μM) for 12 h or 72 h. MACS Dead Cell Removal Kit (Miltenyi Biotec, Gladbach, DEU) was performed according to the manufacturer’s instructions on cells treated for 72 h to increase the viability of the samples before RNA-sequencing. The viability of the samples treated for 12 h was not subjected to Dead Cell Removal as the viability was already sufficient. All samples were washed in PBS with 0.04% BSA (2 × 1 mL). Chromium Next GEM Single Cell 3′ library preparation and RNA-sequencing were performed by the SNP&SEQ Technology Platform (National Genomics Infrastructure (NGI), Science for Life Laboratory, Uppsala University, Sweden).

### scRNA-seq data processing

The single cell data was first processed using Cell Ranger toolkit version 5.0.1 provided by 10 × Genomics, used for demultiplexing, aligning reads to the human reference genome GRCh38, and generating gene-cell unique molecular identifiers (UMIs). Bioinformatic analyses of the CellRanger output was performed using the Seurat package^47^ of the R programming language, with modifications and additions. Cell libraries were considered to be of low quality and filtered out if: (1) fewer than 200 genes were detected; (2) they had a higher mitochondrial content than 25%; (3) they had a lower ribosomal content than 5%. Genes present in fewer than 3 cells were excluded, as were mitochondrial genes and the MALAT1 gene. Gene counts were log-normalized with a scaling factor of 10,000, followed by Z-score transformation for regression of confounding factors and downstream clustering. Doublets were found using DoubletFinder^48^ and subsequently removed from further analyses.

The normalized data was subsequently processed using principal component analysis (PCA), from which the top 50 components were selected for downstream analyses. The Uniform Manifold Approximation & Projected (UMAP)^49^ non-linear method for dimensionality reduction was run on the top 50 principal components, yielding a final embedding of two dimensions. Unsupervised graph clustering using the Louvain method was run using a resolution parameter of 0.2, optimized to yield a coherent cluster distribution in the UMAP. Clusters were manually annotated using known cell type markers and the resulting cell types were used in further analyses.

### scRNA-seq data analysis

Differential expression testing between cases and controls for each previously defined cell type was performed using MAST^50^. A log(fold change) threshold of 0.25 and false discovery rate of 0.01 was used. Differential expression testing between clusters was run in the same manner. Trajectory analyses were run using Slingshot^40^ on subset and re-clustered data, performed in the same manner as described above. Gene selection for trajectory analyses was performed using a random forest classifier^51^ on scaled expression data with default settings, variables were then selected based on mean decrease accuracy. Cell cycle phases were inferred using CellCycleScoring in the Seurat package^47^. All bioinformatic analyses post-CellRanger have been put into a Nextflow-based workflow to facilitate reproducibility, which can be found on GitHub. Analyses can be reproduced with either Docker or Conda, though Docker is the preferred method.

## Supplementary Information

Supplementary Information.

## Data Availability

Data is accessible from the SciLifeLab Data Repository, link: https://doi.org/10.17044/scilifelab.c.5997556 or from the authors. Counts data is available for direct download. To ensure the integrity of the anonymous PBMC donor, the sequence data is only available upon reasonable request. Code is available upon request.
