# Supplementary material for: Single-cell transcriptional pharmacodynamics of trifluridine in a tumor-immune model
Source: Sci Rep. 2022 Jul 13;12:11960. doi: 10.1038/s41598-022-16077-7 (PMC9279337; doi:10.1038/s41598-022-16077-7)
Supplement: Supplementary file 1 — Supplementary Information. [file 41598_2022_16077_MOESM1_ESM.pdf]

Single-cell transcriptional pharmacodynamics of trifluridine in a tumor-immune model

Tove Selvin[1]*, Erik Fasterius[2], Malin Jarvius[1,3], Mårten Fryknäs[1], Rolf Larsson[1] and Claes R Andersson[1]*

[1]Department of Medical Sciences, Uppsala University, 75185 Uppsala, Sweden.
[2]National Bioinformatics Infrastructure Sweden (NBIS), Uppsala University, 75124 Uppsala, Sweden.
[3]Current address: Department of Pharmaceutical Biosciences and Science for Life Laboratory, Uppsala University, Box 591, SE-751 24 Uppsala, Sweden

Supplementary Figure 1

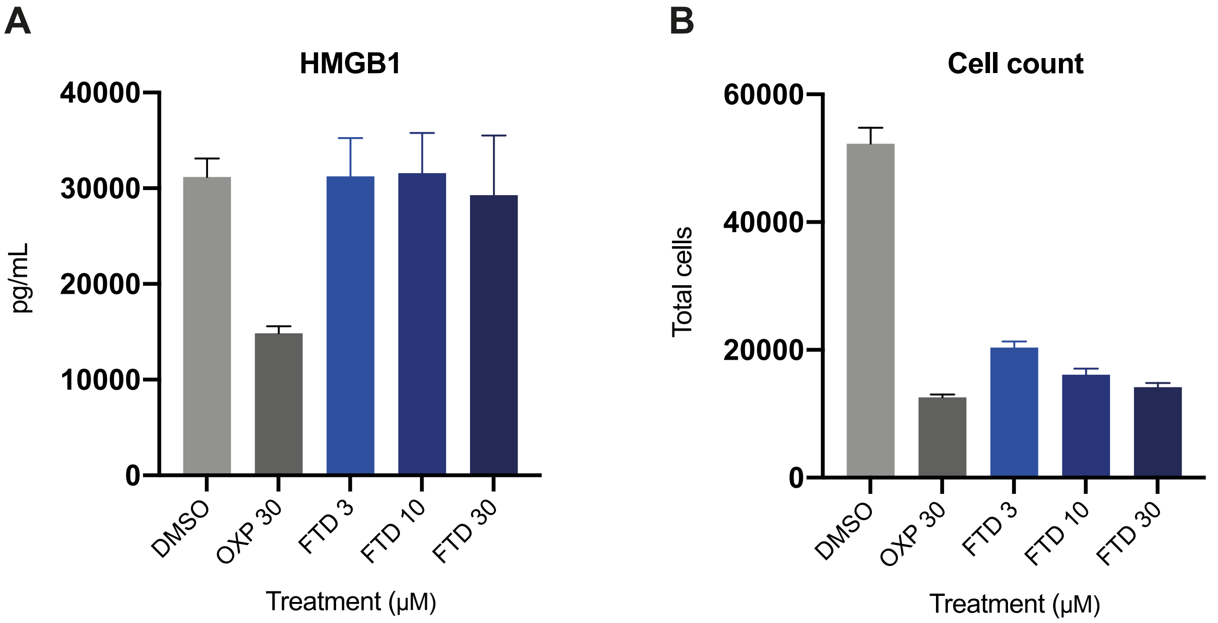

A) HMGB1 measured by ELISA in supernatants of HCT116-GFP monoculture treated for 72 h. B) Cell count performed prior to ELISA after 72 h treatment, used to normalize HMGB1 measurement. Results are shown as mean ± SD from two

Supplementary Figure 2

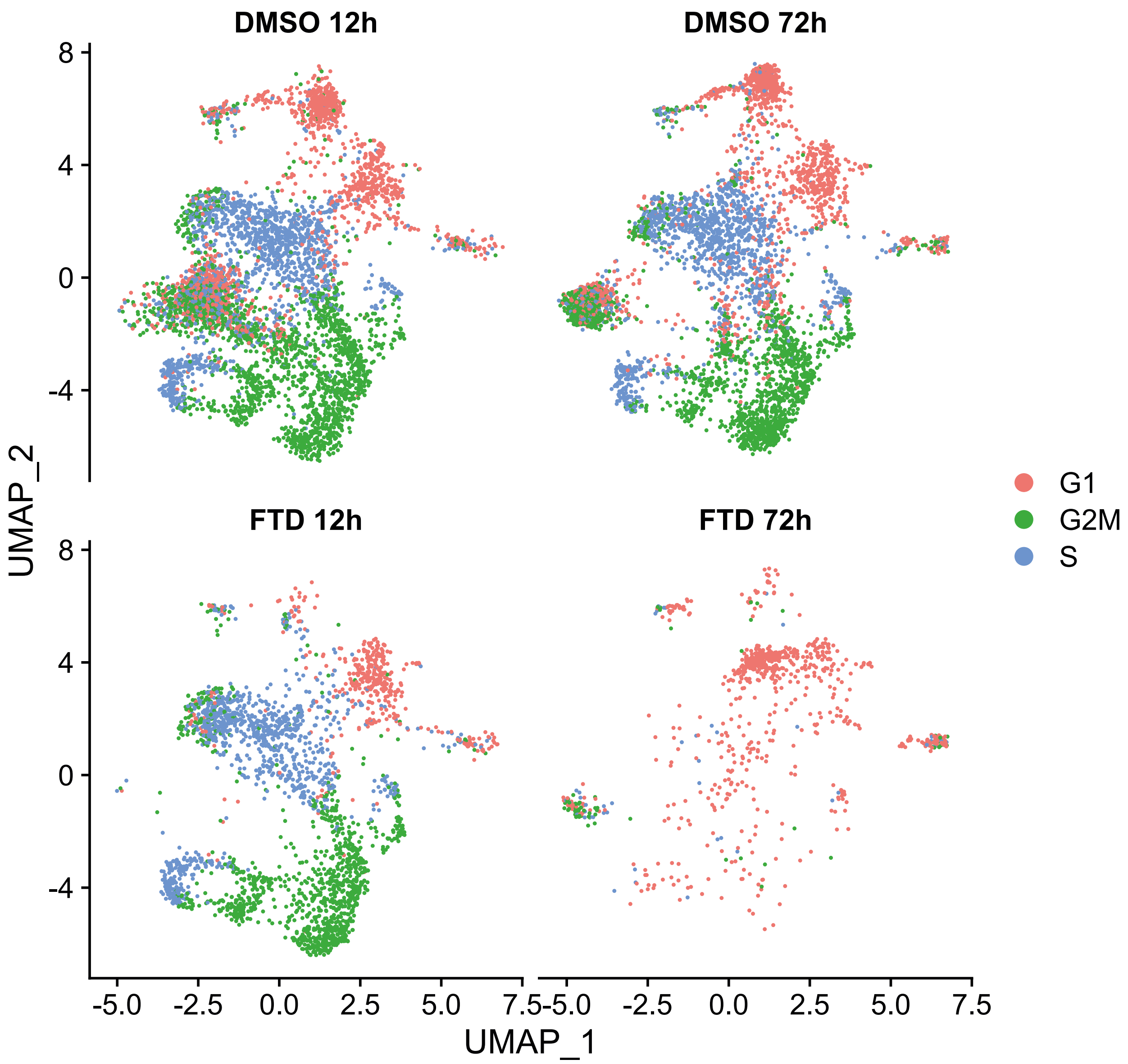

HCT116 from all samples (12h and 72h) projected together but separately from PBMC and colored by cell cycle phase as inferred by Seurat. The most pronounced FTD-induced effect is seen at 72h for which the trajectory analysis was performed.

Supplementary data 1

DEGs HCT116 12h

gene	p_val	avg_log2FC	pct,1	pct,2	p_val_adj
AL627171.2	1.31E-158	0.81	0.686	0.363	3.00E-154
PLCG2	1.04E-94	0.65	0.558	0.324	2.38E-90
HIST1H4C	6.82E-253	0.53	0.944	0.606	1.56E-248
RPS29	1.00E-270	0.46	0.991	0.87	2.29E-266
MTRNR2L1	0	0.44	0.943	0.533	0
ATP5ME	4.64E-265	0.44	0.982	0.686	1.06E-260
MTRNR2L8	6.85E-284	0.44	0.87	0.464	1.56E-279
RPS28	2.69E-204	0.35	0.993	0.913	6.14E-200
RPL37A	3.79E-168	0.34	0.995	0.911	8.65E-164
RPS27	7.40E-163	0.33	0.995	0.911	1.69E-158
MTRNR2L12	0	0.31	0.972	0.599	0
METAP2	1.59E-295	0.30	0.968	0.62	3.63E-291
NDUFB1	1.04E-298	0.29	0.958	0.595	2.37E-294
RPL38	1.40E-136	0.29	0.994	0.872	3.19E-132
RPS21	4.25E-142	0.28	0.995	0.918	9.71E-138
PET100	1.51E-318	0.28	0.968	0.599	3.44E-314
RPL36	6.03E-113	0.26	0.996	0.928	1.38E-108
NAA38	1.09E-310	0.26	0.964	0.602	2.48E-306
EIF5B	2.58E-282	0.25	0.969	0.637	5.88E-278
C4orf48	7.29E-284	0.25	0.977	0.65	1.66E-279
CCL4	1.37E-78	-1.14	0.26	0.207	3.14E-74
B2M	8.54E-194	-0.75	0.986	0.947	1.95E-189
HLA-B	2.09E-271	-0.75	0.964	0.72	4.76E-267
CCL3	3.68E-35	-0.70	0.118	0.096	8.39E-31
HLA-DRA	5.83E-61	-0.68	0.221	0.151	1.33E-56
SNHG25	5.45E-262	-0.57	0.956	0.673	1.24E-257
HLA-A	7.35E-298	-0.56	0.966	0.705	1.68E-293
IFI6	1.22E-164	-0.49	0.81	0.524	2.78E-160
HLA-C	2.39E-299	-0.45	0.973	0.725	5.45E-295
MYC	1.01E-272	-0.45	0.9	0.61	2.30E-268
TMSB4X	1.00E-265	-0.44	0.961	0.7	2.29E-261
HLA-DRB1	4.79E-30	-0.41	0.159	0.111	1.09E-25
CXCL8	7.12E-32	-0.40	0.169	0.106	1.63E-27
CKS2	7.56E-240	-0.36	0.959	0.719	1.73E-235
VIM	8.59E-48	-0.35	0.353	0.229	1.96E-43
SERPINB1	1.14E-172	-0.34	0.805	0.502	2.59E-168
SOD2	1.27E-294	-0.34	0.935	0.602	2.90E-290
IFI30	2.45E-221	-0.32	0.865	0.547	5.59E-217
PSMB9	6.26E-283	-0.31	0.945	0.659	1.43E-278
CDKN1A	5.70E-161	-0.29	0.779	0.482	1.30E-156
ISG20	2.29E-76	-0.29	0.533	0.337	5.22E-72
GBP1	1.27E-19	-0.28	0.215	0.164	2.90E-15
HNRNPA2B1	2.42E-234	-0.28	0.981	0.84	5.53E-230
TAP1	2.17E-312	-0.27	0.95	0.648	4.96E-308
LDHA	8.55E-228	-0.27	0.976	0.825	1.95E-223

ISG15	6.14E-238	-0.26	0.986	0.764	1.40E-233
WARS	2.94E-251	-0.25	0.897	0.571	6.71E-247
MYL12B	3.77E-214	-0.25	0.975	0.815	8.61E-210
HNRNPH1	5.20E-313	-0.25	0.958	0.641	0.00E+00
NFKBIA	1.62E-278	-0.25	0.915	0.588	3.69E-274
HNRNPDL	0	-0.25	0.959	0.653	0
MAD2L1	5.28E-250	-0.25	0.913	0.625	1.20E-245

DEGs HCT116 72h

gene	p_val	avg_log2FC	pct.1	pct.2	p_val_adj
AL627171.2	2.01E-28	2.89	0.518	0.411	4.58E-24
PLCG2	2.87E-55	2.44	0.561	0.305	6.55E-51
MTRNR2L12	7.60E-13	1.72	0.832	0.717	1.73E-08
CDKN1A	4.34E-268	1.54	0.792	0.534	9.90E-264
ISG15	1.92E-220	1.41	0.891	0.857	4.37E-216
S100A6	1.41E-259	1.31	0.953	0.945	3.21E-255
KRT8	3.03E-269	1.22	0.881	0.891	6.92E-265
SERPINB1	3.59E-180	1.19	0.761	0.655	8.18E-176
SFN	6.71E-190	1.16	0.843	0.777	1.53E-185
IFI6	3.75E-158	1.14	0.765	0.724	8.55E-154
SH3BGRL3	8.23E-247	1.14	0.811	0.735	1.88E-242
KRT18	5.17E-238	1.13	0.914	0.917	1.18E-233
KRT19	5.23E-210	1.10	0.887	0.881	1.19E-205
FDXR	1.35E-195	1.10	0.769	0.476	3.09E-191
GDF15	1.14E-203	1.09	0.671	0.155	2.61E-199
S100A10	1.56E-180	1.08	0.861	0.852	3.57E-176
TP53I3	1.58E-219	1.05	0.709	0.209	3.62E-215
RPS27L	1.62E-202	0.99	0.821	0.788	3.70E-198
PLEC	1.85E-106	0.99	0.804	0.648	4.21E-102
AC007952.4	5.45E-19	0.96	0.139	0.108	1.24E-14
GLRX	4.40E-150	0.95	0.739	0.495	1.00E-145
IFI27	2.19E-136	0.95	0.729	0.523	4.99E-132
FTH1	1.08E-148	0.94	0.955	0.975	2.48E-144
ADIRF	3.86E-102	0.94	0.791	0.702	8.81E-98
KRTAP3-1	3.95E-36	0.92	0.139	0.026	9.02E-32
KRTAP2-3	1.13E-67	0.91	0.684	0.483	2.58E-63
MTRNR2L1	1.11E-23	0.91	0.756	0.594	2.53E-19
CYBA	7.57E-201	0.90	0.854	0.793	1.73E-196
CST3	6.33E-183	0.87	0.774	0.717	1.44E-178
FBXO2	4.36E-154	0.82	0.745	0.553	9.96E-150
ISG20	6.29E-105	0.82	0.736	0.596	1.44E-100
IGFBP6	1.66E-86	0.81	0.743	0.575	3.79E-82
EPS8L2	1.01E-133	0.80	0.731	0.343	2.29E-129
SELENOM	1.12E-157	0.78	0.764	0.715	2.56E-153
C19orf33	3.89E-108	0.77	0.806	0.732	8.88E-104
FHL2	1.46E-148	0.75	0.753	0.635	3.33E-144
RHOD	9.12E-138	0.75	0.749	0.598	2.08E-133
CPA4	1.41E-85	0.74	0.639	0.333	3.22E-81

IFI27L2	7.54E-133	0.74	0.769	0.614	1.72E-128
MTRNR2L8	7.20E-25	0.73	0.693	0.499	1.64E-20
S100A4	5.71E-39	0.73	0.68	0.55	1.30E-34
CD59	7.47E-125	0.72	0.774	0.719	1.70E-120
POLD4	4.19E-150	0.72	0.745	0.428	9.56E-146
RHOC	7.93E-139	0.72	0.774	0.721	1.81E-134
SAT1	2.40E-60	0.71	0.799	0.736	5.48E-56
PHLDA3	1.13E-139	0.70	0.738	0.434	2.59E-135
VPS28	3.23E-113	0.69	0.776	0.674	7.38E-109
BTG1	3.81E-120	0.69	0.746	0.544	8.69E-116
PHPT1	1.57E-107	0.68	0.797	0.721	3.57E-103
MYL9	5.59E-113	0.67	0.741	0.538	1.28E-108
EDF1	2.19E-110	0.67	0.844	0.763	5.00E-106
MAP1LC3B	3.81E-102	0.67	0.794	0.724	8.70E-98
LGALS3	8.40E-133	0.67	0.76	0.69	1.92E-128
BLVRB	1.57E-103	0.66	0.792	0.729	3.58E-99
TYMP	3.28E-83	0.66	0.765	0.664	7.48E-79
ANXA2	2.93E-161	0.65	0.865	0.88	6.68E-157
C4orf3	1.55E-118	0.64	0.761	0.691	3.54E-114
IL32	3.12E-39	0.64	0.518	0.283	7.11E-35
ATOX1	1.07E-91	0.63	0.808	0.749	2.44E-87
BBC3	2.63E-121	0.63	0.684	0.298	6.00E-117
CLIC3	1.84E-152	0.63	0.629	0.194	4.19E-148
MYL6	1.16E-152	0.63	0.887	0.871	2.65E-148
CAPG	8.47E-98	0.63	0.77	0.603	1.93E-93
S100A11	4.15E-124	0.63	0.877	0.876	9.46E-120
NEAT1	3.95E-56	0.62	0.783	0.73	9.01E-52
NPDC1	2.72E-107	0.62	0.751	0.57	6.21E-103
HLA-B	7.16E-96	0.61	0.85	0.817	1.63E-91
C9orf16	2.83E-99	0.61	0.786	0.712	6.45E-95
PPIC	3.33E-112	0.61	0.738	0.588	7.60E-108
IFI35	1.12E-74	0.61	0.779	0.714	2.56E-70
ACADVL	3.27E-78	0.61	0.774	0.639	7.46E-74
GRN	5.53E-107	0.60	0.739	0.6	1.26E-102
VAMP8	5.43E-107	0.59	0.769	0.717	1.24E-102
DUSP5	1.21E-52	0.58	0.77	0.646	2.76E-48
PERP	5.08E-90	0.58	0.758	0.685	1.16E-85
S100A13	8.80E-76	0.58	0.799	0.735	2.01E-71
OPTN	7.67E-96	0.58	0.752	0.65	1.75E-91
RRAS	3.11E-92	0.58	0.758	0.64	7.09E-88
CDA	8.14E-94	0.58	0.709	0.494	1.86E-89
PRDX5	1.92E-100	0.57	0.786	0.745	4.38E-96
ATP5IF1	2.69E-73	0.56	0.839	0.771	6.14E-69
HIST1H1C	1.53E-68	0.56	0.69	0.408	3.49E-64
ATP6V0E1	1.77E-91	0.56	0.766	0.721	4.03E-87
TM7SF2	4.62E-193	0.55	0.646	0.158	1.05E-188
COX14	6.04E-93	0.55	0.767	0.682	1.38E-88
GABARAPL2	3.94E-94	0.55	0.761	0.706	9.00E-90

ISCU	3.25E-82	0.55	0.777	0.697	7.42E-78
TIMP1	1.96E-82	0.55	0.776	0.732	4.48E-78
ELOB	8.08E-67	0.55	0.886	0.807	1.85E-62
TRNP1	7.07E-111	0.55	0.709	0.384	1.61E-106
SERTAD1	6.35E-74	0.54	0.741	0.564	1.45E-69
LGALS1	4.16E-49	0.54	0.879	0.87	9.50E-45
TMEM40	7.30E-161	0.54	0.687	0.247	1.67E-156
IFI30	2.91E-92	0.54	0.754	0.673	6.65E-88
LGALS3BP	3.60E-59	0.54	0.8	0.737	8.22E-55
LAMA5	8.11E-68	0.54	0.494	0.197	1.85E-63
CLTB	7.48E-77	0.54	0.781	0.704	1.71E-72
C4orf48	3.34E-38	0.54	0.833	0.747	7.63E-34
FKBP1A	1.10E-93	0.53	0.787	0.754	2.52E-89
GADD45A	1.02E-55	0.53	0.731	0.54	2.32E-51
TRAPPC5	2.43E-73	0.53	0.792	0.713	5.54E-69
TMSB4X	1.71E-24	0.53	0.826	0.776	3.91E-20
ITGA3	8.31E-63	0.52	0.739	0.541	1.90E-58
AK1	1.01E-88	0.52	0.74	0.643	2.31E-84
CAVIN1	9.31E-75	0.52	0.763	0.661	2.12E-70
ITM2B	4.90E-81	0.52	0.765	0.695	1.12E-76
MYL12B	3.80E-117	0.52	0.83	0.829	8.68E-113
CYSTM1	5.95E-87	0.52	0.767	0.699	1.36E-82
BAX	2.97E-76	0.52	0.761	0.672	6.77E-72
RABAC1	1.12E-86	0.51	0.754	0.65	2.56E-82
RBP1	1.09E-31	0.51	0.778	0.736	2.50E-27
SPINT2	1.50E-75	0.51	0.787	0.753	3.42E-71
FAAP20	7.30E-61	0.51	0.781	0.679	1.67E-56
CCND1	4.72E-50	0.50	0.817	0.785	1.08E-45
ANXA11	4.43E-76	0.50	0.751	0.661	1.01E-71
PGLS	1.98E-93	0.49	0.727	0.49	4.52E-89
TRAF4	7.34E-64	0.49	0.747	0.624	1.68E-59
TNIP1	3.24E-52	0.49	0.732	0.536	7.40E-48
STARD10	1.10E-66	0.49	0.732	0.538	2.50E-62
FXYD5	8.66E-64	0.49	0.767	0.7	1.98E-59
HLA-A	4.80E-59	0.49	0.831	0.802	1.09E-54
GUK1	5.26E-62	0.49	0.827	0.78	1.20E-57
SQSTM1	2.15E-43	0.49	0.799	0.737	4.91E-39
SCAND1	1.22E-68	0.48	0.769	0.622	2.79E-64
GSN	9.12E-135	0.48	0.706	0.345	2.08E-130
CAPN1	2.57E-68	0.48	0.757	0.646	5.87E-64
IL18	2.98E-64	0.48	0.741	0.637	6.79E-60
TSPO	2.11E-76	0.48	0.787	0.753	4.80E-72
TRAPPC1	4.32E-87	0.48	0.757	0.706	9.85E-83
GSTK1	4.60E-70	0.48	0.754	0.652	1.05E-65
SQOR	3.88E-104	0.48	0.71	0.4	8.85E-100
MXD4	1.47E-126	0.47	0.707	0.306	3.35E-122
PSAP	2.10E-81	0.47	0.747	0.599	4.78E-77
DHRS7	1.33E-83	0.47	0.745	0.629	3.03E-79

PMAIP1	8.10E-48	0.47	0.772	0.715	1.85E-43
GSDMD	3.49E-50	0.47	0.761	0.635	7.96E-46
WDR45	3.53E-128	0.47	0.722	0.359	8.05E-124
REX1BD	4.21E-49	0.47	0.773	0.667	9.62E-45
AREG	6.45E-24	0.46	0.807	0.782	1.47E-19
TPGS1	4.54E-62	0.46	0.736	0.514	1.04E-57
NDUFA11	1.79E-70	0.46	0.832	0.777	4.09E-66
CORO1B	9.53E-91	0.46	0.745	0.618	2.18E-86
HLA-C	1.69E-64	0.46	0.838	0.81	3.87E-60
EHD1	4.64E-68	0.45	0.699	0.461	1.06E-63
PLK2	1.52E-58	0.45	0.734	0.551	3.46E-54
NDUFB7	1.48E-40	0.45	0.827	0.748	3.38E-36
TMSB10	8.02E-81	0.45	0.986	0.986	1.83E-76
HIST1H2AC	2.16E-160	0.44	0.643	0.184	4.92E-156
ACAA1	2.82E-71	0.44	0.727	0.527	6.44E-67
ITGA2	1.08E-53	0.44	0.71	0.49	2.46E-49
RRAD	2.32E-132	0.44	0.403	0.064	5.30E-128
GAS6	1.06E-38	0.44	0.722	0.586	2.42E-34
COPE	1.20E-55	0.44	0.803	0.731	2.73E-51
SMURF2	1.04E-52	0.44	0.746	0.643	2.37E-48
PLXNB2	2.57E-45	0.44	0.69	0.434	5.87E-41
UQCR11	3.70E-66	0.44	0.827	0.781	8.44E-62
TUBA1A	4.51E-40	0.44	0.709	0.543	1.03E-35
TMEM59	2.00E-81	0.44	0.749	0.684	4.56E-77
SERF2	1.87E-88	0.43	0.868	0.837	4.27E-84
AHNAK	2.47E-38	0.43	0.764	0.64	5.64E-34
NDUFA13	4.56E-42	0.43	0.87	0.789	1.04E-37
CD63	1.41E-70	0.43	0.807	0.792	3.22E-66
GNAI2	1.27E-59	0.43	0.759	0.682	2.91E-55
PHLDA2	3.35E-44	0.43	0.867	0.827	7.64E-40
TPM2	1.83E-41	0.43	0.845	0.802	4.18E-37
PLIN3	1.05E-60	0.43	0.77	0.719	2.40E-56
IFITM3	2.23E-49	0.42	0.816	0.78	5.10E-45
ORMDL2	2.01E-69	0.42	0.723	0.561	4.58E-65
CCS	4.16E-85	0.42	0.718	0.397	9.48E-81
PPP1R12C	3.49E-49	0.42	0.503	0.243	7.96E-45
POLR2L	3.52E-32	0.42	0.881	0.838	8.03E-28
KHDC1L	7.96E-83	0.42	0.649	0.341	1.82E-78
ANXA4	1.91E-82	0.42	0.719	0.51	4.36E-78
SDSL	7.25E-91	0.41	0.713	0.436	1.65E-86
CMBL	5.67E-68	0.41	0.711	0.487	1.29E-63
CSTB	6.39E-62	0.41	0.794	0.767	1.46E-57
RPS19	1.10E-78	0.41	0.981	0.988	2.50E-74
MYL12A	3.00E-55	0.41	0.792	0.758	6.86E-51
C1orf122	8.85E-51	0.41	0.761	0.686	2.02E-46
TXNDC17	1.44E-47	0.41	0.824	0.775	3.30E-43
GABARAP	7.30E-61	0.41	0.769	0.729	1.67E-56
PNRC1	4.77E-167	0.41	0.664	0.186	1.09E-162

TIMP2	3.77E-57	0.41	0.714	0.51	8.60E-53
RNASEK	3.15E-66	0.40	0.781	0.755	7.18E-62
TMEM219	3.47E-58	0.40	0.736	0.595	7.91E-54
MGST3	1.47E-51	0.40	0.769	0.723	3.36E-47
ARPC2	8.25E-58	0.40	0.8	0.768	1.88E-53
ATF3	6.99E-68	0.40	0.711	0.418	1.60E-63
ARPC1B	1.28E-41	0.40	0.804	0.754	2.92E-37
TMEM14C	8.63E-61	0.40	0.75	0.679	1.97E-56
UROS	2.96E-64	0.40	0.704	0.529	6.76E-60
PLCD3	2.11E-115	0.39	0.682	0.296	4.81E-111
RAB25	3.55E-57	0.39	0.72	0.597	8.10E-53
PSME2	3.47E-45	0.39	0.857	0.828	7.92E-41
ATP6V1F	1.93E-61	0.39	0.773	0.741	4.40E-57
JUP	1.11E-58	0.39	0.722	0.484	2.53E-54
PNPLA2	6.07E-50	0.39	0.719	0.489	1.39E-45
GPRC5A	2.22E-53	0.39	0.74	0.634	5.06E-49
FLII	5.86E-32	0.39	0.736	0.578	1.34E-27
SIL1	1.47E-62	0.39	0.69	0.426	3.37E-58
B2M	6.22E-45	0.38	0.913	0.926	1.42E-40
LSR	6.49E-61	0.38	0.749	0.682	1.48E-56
NBDY	3.94E-60	0.38	0.75	0.678	9.00E-56
APRT	2.00E-50	0.38	0.827	0.793	4.57E-46
PDLIM1	7.74E-53	0.38	0.733	0.635	1.77E-48
CCL5	2.91E-49	0.38	0.301	0.097	6.65E-45
NUDT22	7.92E-56	0.38	0.733	0.566	1.81E-51
BLOC1S2	2.35E-42	0.38	0.754	0.655	5.37E-38
TRIAP1	1.64E-53	0.38	0.749	0.657	3.75E-49
LAMB3	2.10E-68	0.38	0.652	0.355	4.80E-64
YIPF3	7.36E-70	0.38	0.739	0.573	1.68E-65
TCEAL4	1.22E-43	0.38	0.72	0.529	2.79E-39
JOSD2	1.06E-45	0.38	0.74	0.59	2.43E-41
LRP10	1.02E-78	0.38	0.709	0.44	2.33E-74
CDC37	2.48E-27	0.37	0.825	0.735	5.65E-23
UBL5	1.51E-47	0.37	0.841	0.788	3.44E-43
MMP24OS	2.24E-80	0.37	0.712	0.452	5.12E-76
DAPK3	4.88E-35	0.37	0.706	0.498	1.11E-30
GAMT	1.93E-22	0.37	0.738	0.627	4.40E-18
MARCH2	1.57E-93	0.37	0.671	0.327	3.57E-89
NFKB2	6.09E-15	0.37	0.756	0.628	1.39E-10
IER5	4.23E-21	0.37	0.685	0.52	9.66E-17
AP2A1	2.35E-41	0.37	0.724	0.529	5.36E-37
ATP6V1D	2.84E-56	0.37	0.753	0.629	6.47E-52
EID1	3.29E-39	0.37	0.767	0.692	7.52E-35
PIEZO1	2.53E-27	0.37	0.678	0.477	5.77E-23
FADS3	4.74E-78	0.37	0.704	0.449	1.08E-73
DRAP1	4.85E-40	0.37	0.811	0.741	1.11E-35
CCDC186	3.85E-54	0.37	0.713	0.477	8.78E-50
NEDD4L	4.52E-80	0.36	0.694	0.386	1.03E-75

BORCS7	6.45E-47	0.36	0.702	0.477	1.47E-42
CCDC57	4.47E-43	0.36	0.754	0.607	1.02E-38
MDK	4.19E-28	0.36	0.794	0.737	9.57E-24
MDM2	5.48E-46	0.36	0.696	0.473	1.25E-41
ROMO1	1.85E-19	0.36	0.806	0.711	4.22E-15
MIEN1	3.34E-35	0.36	0.756	0.66	7.62E-31
DUSP14	3.82E-66	0.36	0.733	0.603	8.72E-62
CLU	5.95E-23	0.36	0.74	0.652	1.36E-18
CST6	3.52E-47	0.36	0.754	0.646	8.03E-43
S100A16	4.70E-37	0.36	0.766	0.723	1.07E-32
DPP7	1.49E-19	0.36	0.773	0.671	3.41E-15
SYTL1	2.68E-132	0.36	0.546	0.141	6.12E-128
POLR3GL	5.19E-65	0.36	0.687	0.488	1.18E-60
NME3	6.20E-31	0.36	0.759	0.662	1.41E-26
PLK3	3.48E-52	0.35	0.705	0.454	7.94E-48
BTG2	1.04E-138	0.35	0.579	0.156	2.37E-134
RSRP1	4.45E-32	0.35	0.541	0.324	1.02E-27
SLFN5	1.42E-105	0.35	0.663	0.281	3.23E-101
SP100	1.06E-44	0.35	0.754	0.683	2.42E-40
SDC4	2.11E-21	0.35	0.719	0.59	4.81E-17
SNHG12	1.24E-07	0.35	0.622	0.519	0.0028
NDUFA4	2.13E-45	0.35	0.819	0.791	4.87E-41
NORAD	8.66E-52	0.35	0.714	0.464	1.98E-47
HMOX2	5.60E-45	0.35	0.756	0.661	1.28E-40
FAM50A	6.55E-49	0.35	0.739	0.644	1.49E-44
ACTR10	8.26E-42	0.35	0.736	0.582	1.89E-37
KLK8	6.74E-42	0.35	0.697	0.501	1.54E-37
CTSB	1.98E-48	0.35	0.746	0.604	4.53E-44
MAP2K3	1.45E-39	0.35	0.758	0.667	3.31E-35
ETFB	1.96E-32	0.34	0.793	0.715	4.48E-28
GIPC1	7.90E-46	0.34	0.754	0.675	1.80E-41
ABCA7	1.90E-13	0.34	0.677	0.539	4.33E-09
LMTK3	4.18E-59	0.34	0.693	0.455	9.55E-55
APOBEC3C	1.31E-111	0.34	0.67	0.269	2.99E-107
FKBP8	1.67E-44	0.34	0.76	0.678	3.82E-40
CYB5A	8.98E-35	0.34	0.72	0.605	2.05E-30
FSTL3	3.06E-67	0.34	0.632	0.326	6.97E-63
MAP2K2	6.00E-36	0.34	0.786	0.713	1.37E-31
CORO1A	8.88E-20	0.34	0.243	0.115	2.03E-15
SSR4	1.71E-39	0.34	0.776	0.733	3.90E-35
GSS	6.09E-45	0.33	0.732	0.551	1.39E-40
CAV2	6.60E-46	0.33	0.754	0.7	1.51E-41
YPEL5	2.75E-153	0.33	0.671	0.212	6.27E-149
FAM177A1	9.82E-48	0.33	0.751	0.682	2.24E-43
ALDH1A3	4.29E-41	0.33	0.618	0.386	9.78E-37
MYH9	4.46E-24	0.33	0.759	0.628	1.02E-19
BSG	8.52E-35	0.33	0.788	0.737	1.94E-30
SPINT1	1.20E-44	0.33	0.729	0.59	2.74E-40

BRK1	6.65E-44	0.33	0.758	0.704	1.52E-39
ARL6IP5	5.32E-46	0.33	0.734	0.604	1.21E-41
PFDN5	3.73E-41	0.33	0.845	0.793	8.51E-37
CXCL1	8.82E-65	0.33	0.387	0.126	2.01E-60
ARF5	1.62E-42	0.33	0.739	0.639	3.70E-38
CXCL16	5.62E-47	0.33	0.711	0.574	1.28E-42
ATP6V1G1	2.22E-42	0.33	0.774	0.727	5.07E-38
SAMD9	4.81E-45	0.33	0.673	0.463	1.10E-40
CCDC90B	1.12E-48	0.33	0.724	0.549	2.57E-44
CIB1	1.24E-32	0.33	0.773	0.705	2.84E-28
ACTR2	3.16E-26	0.33	0.759	0.632	7.22E-22
INTS1	5.84E-20	0.33	0.671	0.498	1.33E-15
TUBB2A	1.14E-28	0.33	0.745	0.653	2.60E-24
FBXL6	9.31E-24	0.33	0.712	0.536	2.12E-19
OSTF1	1.18E-40	0.33	0.729	0.556	2.69E-36
STK17A	6.06E-46	0.33	0.75	0.605	1.38E-41
EEF1A2	2.99E-18	0.32	0.792	0.716	6.82E-14
HES2	1.60E-147	0.32	0.582	0.15	3.64E-143
RNF181	4.00E-41	0.32	0.758	0.683	9.14E-37
COMMD5	6.60E-36	0.32	0.729	0.552	1.51E-31
CCNDBP1	9.10E-64	0.32	0.713	0.435	2.08E-59
MSLN	2.62E-95	0.32	0.492	0.151	5.98E-91
TLE5	2.00E-26	0.32	0.764	0.657	4.55E-22
TMC6	1.10E-81	0.32	0.664	0.318	2.52E-77
HIST1H2BD	1.03E-256	0.32	0.559	0.053	2.36E-252
RPL13	5.66E-49	0.32	0.984	0.994	1.29E-44
ATP6AP2	2.42E-39	0.32	0.722	0.599	5.51E-35
SYNGR2	1.63E-21	0.32	0.751	0.671	3.72E-17
ZNHIT1	8.59E-39	0.32	0.771	0.709	1.96E-34
BIRC3	1.05E-21	0.32	0.719	0.606	2.39E-17
TSC22D4	2.59E-49	0.32	0.671	0.402	5.92E-45
IFT20	5.71E-55	0.32	0.717	0.525	1.30E-50
NABP1	3.63E-41	0.32	0.717	0.521	8.28E-37
C15orf48	3.90E-44	0.31	0.287	0.098	8.90E-40
IFITM2	2.39E-31	0.31	0.759	0.668	5.44E-27
BEX3	4.61E-35	0.31	0.754	0.672	1.05E-30
SLC50A1	6.61E-50	0.31	0.725	0.503	1.51E-45
DUS1L	4.44E-15	0.31	0.766	0.673	1.01E-10
LGMN	4.67E-69	0.31	0.706	0.447	1.06E-64
TWF2	1.78E-33	0.31	0.739	0.617	4.07E-29
TMEM238	1.27E-43	0.31	0.682	0.446	2.90E-39
GNAS	2.74E-38	0.31	0.827	0.79	6.25E-34
CAPN2	2.02E-34	0.31	0.75	0.659	4.62E-30
BST2	1.20E-23	0.31	0.677	0.575	2.74E-19
SLC25A37	8.05E-25	0.31	0.736	0.586	1.84E-20
HLA-DRB1	2.95E-09	0.31	0.638	0.526	6.73E-05
REEP5	5.05E-40	0.31	0.772	0.726	1.15E-35
MSRB2	1.58E-78	0.31	0.693	0.394	3.59E-74

MFSD10	6.05E-32	0.31	0.753	0.647	1.38E-27
FBXW5	3.93E-21	0.31	0.744	0.613	8.97E-17
SHFL	1.33E-51	0.31	0.692	0.445	3.03E-47
GCC2	1.29E-44	0.31	0.712	0.485	2.94E-40
TMEM50A	1.15E-43	0.31	0.759	0.706	2.63E-39
ETHE1	2.72E-52	0.31	0.726	0.618	6.22E-48
MIB2	3.36E-16	0.31	0.769	0.673	7.66E-12
RRBP1	5.45E-15	0.31	0.756	0.647	1.24E-10
LAMTOR2	4.48E-31	0.31	0.747	0.657	1.02E-26
MSX1	1.19E-16	0.31	0.729	0.609	2.73E-12
ERCC1	2.49E-29	0.31	0.739	0.607	5.69E-25
CHMP2A	4.43E-44	0.31	0.751	0.686	1.01E-39
BIK	3.90E-37	0.31	0.702	0.493	8.90E-33
TAX1BP1	8.51E-34	0.31	0.756	0.668	1.94E-29
CCNI	2.42E-40	0.31	0.758	0.686	5.51E-36
DUSP4	1.97E-39	0.31	0.686	0.468	4.49E-35
TP53INP1	2.85E-258	0.31	0.499	0.032	6.50E-254
PINK1	1.78E-76	0.30	0.686	0.386	4.07E-72
CTSD	5.82E-37	0.30	0.72	0.58	1.33E-32
NEDD8	1.54E-37	0.30	0.808	0.765	3.53E-33
PTTG1IP	8.48E-35	0.30	0.722	0.543	1.94E-30
SERINC2	2.13E-34	0.30	0.705	0.535	4.86E-30
ARRDC1	2.68E-89	0.30	0.671	0.328	6.11E-85
OCIAD2	2.12E-43	0.30	0.746	0.702	4.83E-39
EFHD2	9.44E-25	0.30	0.753	0.66	2.15E-20
TST	2.96E-90	0.30	0.673	0.314	6.74E-86
CAST	1.85E-22	0.30	0.772	0.7	4.23E-18
NT5E	4.64E-46	0.30	0.672	0.44	1.06E-41
MAN2B1	1.31E-103	0.30	0.566	0.191	3.00E-99
PKM	1.30E-31	0.30	0.86	0.842	2.97E-27
ZNF655	2.43E-43	0.30	0.72	0.508	5.54E-39
SH3GLB1	8.87E-36	0.30	0.75	0.642	2.02E-31
STXBP2	3.01E-50	0.30	0.716	0.472	6.88E-46
PDCD6	1.16E-37	0.30	0.771	0.715	2.65E-33
SERPINB5	6.99E-131	0.30	0.595	0.179	1.60E-126
CREB3	1.08E-45	0.30	0.73	0.549	2.47E-41
MXRA7	5.50E-42	0.30	0.722	0.537	1.26E-37
PPFIBP1	3.99E-46	0.30	0.704	0.473	9.11E-42
ITGB4	6.23E-74	0.30	0.666	0.338	1.42E-69
FXYD3	2.59E-151	0.29	0.411	0.054	5.90E-147
ARF4	5.51E-28	0.29	0.774	0.719	1.26E-23
BSCL2	2.79E-37	0.29	0.713	0.562	6.36E-33
VIM	3.51E-12	0.29	0.448	0.318	8.01E-08
RHOF	5.12E-43	0.29	0.739	0.626	1.17E-38
RPL28	3.17E-24	0.29	0.965	0.947	7.24E-20
ANGPTL4	2.03E-68	0.29	0.455	0.168	4.63E-64
FIS1	7.05E-35	0.29	0.769	0.697	1.61E-30
SEC61B	6.70E-31	0.29	0.799	0.757	1.53E-26

DUSP1	1.29E-30	0.29	0.649	0.465	2.95E-26
AKR1A1	5.66E-24	0.29	0.752	0.663	1.29E-19
QSOX1	3.53E-66	0.29	0.664	0.359	8.05E-62
ATP6V0C	1.32E-30	0.29	0.772	0.722	3.01E-26
ARPC3	6.52E-26	0.29	0.792	0.746	1.49E-21
VAMP2	4.11E-55	0.29	0.698	0.426	9.37E-51
RRP12	2.13E-17	0.29	0.612	0.449	4.87E-13
TMED4	4.12E-42	0.29	0.722	0.555	9.40E-38
CITED2	7.04E-80	0.29	0.59	0.259	1.61E-75
DDIT4	1.78E-52	0.29	0.389	0.148	4.05E-48
ZNF428	1.05E-28	0.29	0.727	0.564	2.41E-24
FLNA	2.44E-22	0.29	0.749	0.647	5.56E-18
PDLIM2	2.79E-36	0.29	0.713	0.525	6.37E-32
ZMAT3	2.73E-138	0.29	0.643	0.203	6.23E-134
TMED3	4.99E-27	0.29	0.719	0.6	1.14E-22
HES4	6.26E-08	0.29	0.765	0.672	0.0014
CHMP1B	5.20E-66	0.29	0.694	0.401	1.19E-61
C19orf53	2.80E-21	0.29	0.819	0.761	6.38E-17
CCN1	6.95E-24	0.29	0.692	0.574	1.59E-19
SLC25A22	7.33E-16	0.29	0.724	0.619	1.67E-11
WDR1	4.30E-27	0.29	0.765	0.677	9.82E-23
EVA1B	4.13E-40	0.29	0.702	0.508	9.43E-36
JUN	1.02E-18	0.29	0.716	0.553	2.34E-14
SEC61A1	9.94E-15	0.28	0.732	0.627	2.27E-10
SMIM14	6.60E-142	0.28	0.631	0.191	1.51E-137
NCOA4	3.73E-35	0.28	0.722	0.518	8.52E-31
YBX3	1.42E-25	0.28	0.799	0.763	3.25E-21
KIFC2	7.30E-58	0.28	0.448	0.179	1.67E-53
LCN2	1.84E-20	0.28	0.21	0.091	4.20E-16
PLSCR3	8.93E-75	0.28	0.632	0.296	2.04E-70
SEPHS2	3.55E-28	0.28	0.727	0.585	8.10E-24
ISYNA1	6.48E-25	0.28	0.71	0.543	1.48E-20
RAC2	3.53E-44	0.28	0.724	0.554	8.06E-40
SLC52A2	2.14E-19	0.28	0.765	0.681	4.88E-15
PROCR	3.50E-24	0.28	0.729	0.633	7.99E-20
PPP1R14B-AS	3.20E-117	0.28	0.655	0.252	7.30E-113
TIMMDC1	1.90E-33	0.28	0.737	0.639	4.35E-29
TRABD	7.14E-24	0.28	0.739	0.585	1.63E-19
RALA	1.97E-33	0.28	0.74	0.649	4.50E-29
MVP	1.77E-21	0.28	0.741	0.631	4.03E-17
COMT	2.30E-46	0.28	0.737	0.638	5.25E-42
CD99	2.04E-42	0.28	0.714	0.585	4.66E-38
AKAP8L	3.76E-18	0.28	0.739	0.587	8.58E-14
CRIP1	8.05E-09	0.28	0.793	0.728	0.0002
CLDN7	9.26E-19	0.28	0.744	0.673	2.11E-14
MIF	2.19E-43	0.28	0.925	0.92	4.99E-39
NOSIP	3.63E-24	0.27	0.79	0.713	8.29E-20
CNKSR1	3.49E-83	0.27	0.575	0.231	7.96E-79

TBCB	5.98E-32	0.27	0.754	0.67	1.36E-27
RTL8C	5.88E-69	0.27	0.686	0.386	1.34E-64
DDX60L	2.66E-32	0.27	0.645	0.443	6.08E-28
EIF6	2.06E-17	0.27	0.798	0.739	4.71E-13
RFNG	8.82E-50	0.27	0.669	0.406	2.01E-45
TGFB1	6.85E-26	0.27	0.705	0.536	1.56E-21
CRIP2	1.61E-84	0.27	0.603	0.263	3.68E-80
AHNAK2	2.31E-140	0.27	0.524	0.118	5.27E-136
TAF7	9.61E-24	0.27	0.734	0.569	2.19E-19
CD81	4.96E-27	0.27	0.784	0.76	1.13E-22
SP110	9.27E-36	0.27	0.71	0.564	2.12E-31
CCL3	4.94E-39	0.27	0.389	0.177	1.13E-34
PSME1	1.46E-24	0.27	0.804	0.767	3.34E-20
MICAL1	4.20E-85	0.27	0.449	0.136	9.59E-81
C18orf32	2.06E-39	0.27	0.743	0.676	4.71E-35
BIRC6	1.34E-20	0.27	0.662	0.485	3.05E-16
TNFRSF10B	1.56E-51	0.27	0.711	0.495	3.57E-47
LLGL2	7.07E-26	0.27	0.726	0.572	1.61E-21
ESPN	2.98E-78	0.27	0.652	0.311	6.80E-74
RRM2B	8.10E-76	0.27	0.675	0.346	1.85E-71
EXOC7	1.24E-60	0.27	0.686	0.388	2.84E-56
DLGAP4	5.80E-74	0.27	0.687	0.368	1.32E-69
RNF7	5.79E-26	0.27	0.773	0.715	1.32E-21
PPP1R16A	1.94E-33	0.27	0.642	0.415	4.42E-29
TRIM8	5.97E-38	0.27	0.697	0.478	1.36E-33
DNPH1	5.48E-29	0.27	0.767	0.678	1.25E-24
SERPINB6	2.86E-30	0.27	0.746	0.65	6.53E-26
PKN1	3.26E-26	0.27	0.745	0.622	7.44E-22
SUPT4H1	8.50E-31	0.26	0.754	0.684	1.94E-26
CERCAM	4.25E-123	0.26	0.611	0.2	9.70E-119
SERPING1	5.28E-109	0.26	0.503	0.145	1.21E-104
NIPAL3	4.62E-139	0.26	0.593	0.165	1.06E-134
TRADD	1.58E-59	0.26	0.697	0.429	3.62E-55
TMEM208	1.59E-19	0.26	0.767	0.689	3.62E-15
CYHR1	2.45E-69	0.26	0.667	0.346	5.60E-65
AHR	1.52E-26	0.26	0.431	0.243	3.47E-22
ANXA5	3.65E-14	0.26	0.788	0.725	8.32E-10
WWC1	1.35E-38	0.26	0.729	0.551	3.08E-34
TNFRSF6B	2.32E-17	0.26	0.612	0.469	5.29E-13
ATP5F1E	4.09E-26	0.26	0.886	0.852	9.34E-22
DYNLT1	2.95E-29	0.26	0.769	0.747	6.73E-25
SEC14L1	3.26E-48	0.26	0.667	0.398	7.44E-44
TERF2IP	3.42E-29	0.26	0.739	0.599	7.81E-25
NDUFV3	1.96E-16	0.26	0.74	0.634	4.48E-12
STX12	2.02E-51	0.26	0.689	0.423	4.61E-47
RNPEPL1	1.28E-57	0.26	0.653	0.363	2.93E-53
SOCS1	3.58E-45	0.26	0.548	0.29	8.17E-41
ILK	2.55E-34	0.26	0.739	0.643	5.81E-30

LASP1	6.57E-37	0.26	0.698	0.477	1.50E-32
CSNK1G2	1.76E-14	0.26	0.709	0.565	4.03E-10
SMIM26	1.21E-26	0.26	0.76	0.684	2.76E-22
TENT5A	1.77E-29	0.25	0.627	0.418	4.05E-25
BLCAP	3.37E-92	0.25	0.682	0.313	7.69E-88
ZNFX1	1.93E-39	0.25	0.719	0.525	4.40E-35
MROH6	2.08E-66	0.25	0.626	0.311	4.74E-62
PCYT2	1.11E-31	0.25	0.703	0.5	2.54E-27
UBXN4	9.53E-22	0.25	0.769	0.68	2.18E-17
MRPL41	7.08E-14	0.25	0.828	0.759	1.62E-09
HEXIM1	5.96E-41	0.25	0.663	0.411	1.36E-36
CSNK2B	1.15E-28	0.25	0.772	0.708	2.63E-24
C11orf68	1.61E-51	0.25	0.697	0.436	3.67E-47
ZMIZ2	3.13E-62	0.25	0.584	0.28	7.15E-58
LYPLA2	1.72E-29	0.25	0.736	0.581	3.92E-25
PSENEN	7.68E-34	0.25	0.722	0.596	1.75E-29
HES1	2.03E-15	0.25	0.281	0.158	4.62E-11
IRF7	7.47E-72	0.25	0.596	0.272	1.70E-67
SIPA1	1.44E-45	0.25	0.713	0.48	3.28E-41
ID3	4.28E-14	0.25	0.687	0.624	9.76E-10
FBXO22	6.07E-21	0.25	0.751	0.648	1.39E-16
HEXD	6.64E-42	0.25	0.649	0.397	1.51E-37
MTIF3	2.15E-31	0.25	0.702	0.522	4.91E-27
SOX9	4.07E-62	0.25	0.642	0.336	9.28E-58
TSG101	1.60E-35	0.25	0.737	0.623	3.65E-31
CCNB1	3.87E-196	-1.45	0.435	0.694	8.84E-192
TUBA1B	0	-1.28	0.853	0.868	0
HMGB2	9.92E-218	-1.26	0.488	0.715	2.26E-213
H2AFZ	0	-1.25	0.848	0.854	0
CDC20	1.85E-197	-1.24	0.243	0.68	4.22E-193
HIST1H4C	5.16E-131	-1.21	0.543	0.687	1.18E-126
HMGB1	0	-1.20	0.872	0.857	0
UBE2C	4.01E-135	-1.16	0.405	0.697	9.16E-131
MAD2L1	2.26E-233	-1.15	0.327	0.681	5.15E-229
HSPD1	0	-1.10	0.86	0.863	0
HSPE1	0	-1.09	0.864	0.864	0
BIRC5	3.21E-191	-1.09	0.282	0.689	7.32E-187
RANBP1	0	-1.06	0.778	0.777	0
KPNA2	4.36E-208	-1.05	0.767	0.748	9.96E-204
UBE2S	0	-1.04	0.841	0.816	0
NASP	0	-1.02	0.71	0.714	0
PTTG1	4.16E-271	-0.99	0.678	0.718	9.48E-267
CKS1B	1.56E-285	-0.95	0.692	0.721	3.57E-281
TUBA1C	6.11E-298	-0.95	0.806	0.796	1.40E-293
HSP90AB1	2.42E-257	-0.93	0.917	0.936	5.53E-253
TUBB	1.07E-313	-0.93	0.812	0.806	0.00E+00
UBE2T	4.84E-174	-0.93	0.463	0.67	1.11E-169
PCLAF	4.46E-155	-0.91	0.448	0.69	1.02E-150

NCL	2.08E-269	-0.91	0.864	0.818	4.75E-265
MKI67	2.03E-144	-0.91	0.261	0.666	4.62E-140
SRSF3	0	-0.90	0.805	0.771	0
NPM1	0	-0.88	0.88	0.908	0
PTMA	2.58E-279	-0.88	0.959	0.961	5.89E-275
PTGES3	0	-0.86	0.805	0.798	0
CENPF	1.60E-124	-0.86	0.267	0.648	3.66E-120
CACYBP	7.76E-237	-0.86	0.751	0.728	1.77E-232
CDK1	1.10E-119	-0.85	0.193	0.601	2.51E-115
CKS2	5.48E-148	-0.85	0.779	0.747	1.25E-143
HNRNPA1	1.43E-307	-0.85	0.783	0.753	3.26E-303
MCM7	4.50E-150	-0.83	0.422	0.67	1.03E-145
ANP32B	6.40E-255	-0.81	0.776	0.737	1.46E-250
HSP90AA1	1.23E-175	-0.81	0.919	0.958	2.80E-171
TYMS	5.75E-218	-0.80	0.663	0.704	1.31E-213
CDKN3	7.38E-125	-0.80	0.357	0.65	1.68E-120
SNRPG	4.95E-305	-0.80	0.806	0.789	1.13E-300
DLGAP5	4.53E-179	-0.80	0.092	0.586	1.03E-174
LSM5	7.80E-206	-0.80	0.749	0.725	1.78E-201
PBK	3.72E-156	-0.79	0.208	0.645	8.50E-152
NUSAP1	1.05E-112	-0.79	0.325	0.628	2.40E-108
HMMR	1.88E-147	-0.78	0.13	0.577	4.30E-143
GINS2	1.30E-132	-0.78	0.301	0.642	2.96E-128
STMN1	2.67E-214	-0.75	0.773	0.75	6.09E-210
HMGN2	4.11E-240	-0.74	0.776	0.744	9.39E-236
SRSF7	2.12E-211	-0.74	0.784	0.736	4.83E-207
NCAPG	7.17E-183	-0.73	0.108	0.611	1.64E-178
RRM2	2.56E-150	-0.73	0.243	0.664	5.85E-146
HNRNPD	2.25E-207	-0.72	0.766	0.715	5.14E-203
HSPA8	5.46E-189	-0.72	0.868	0.868	1.25E-184
DTYMK	1.46E-160	-0.72	0.687	0.685	3.34E-156
SNRPD1	4.89E-239	-0.72	0.806	0.78	1.12E-234
ZWINT	1.11E-120	-0.71	0.29	0.645	2.53E-116
ATAD2	1.45E-122	-0.71	0.398	0.638	3.31E-118
SNRPB	1.09E-238	-0.71	0.804	0.789	2.49E-234
ANP32E	1.01E-168	-0.71	0.664	0.674	2.31E-164
RBMX	2.65E-110	-0.71	0.704	0.689	6.05E-106
HNRNPA2B1	1.24E-271	-0.71	0.847	0.829	2.82E-267
LSM3	2.33E-172	-0.70	0.778	0.749	5.31E-168
AURKA	4.57E-83	-0.69	0.313	0.559	1.04E-78
SET	1.82E-204	-0.69	0.82	0.793	4.16E-200
PRC1	1.42E-136	-0.69	0.246	0.631	3.25E-132
TMPO	9.60E-121	-0.69	0.434	0.645	2.19E-116
SNRPF	1.12E-236	-0.69	0.794	0.776	2.56E-232
RAD21	1.76E-126	-0.68	0.713	0.676	4.01E-122
PAICS	2.35E-175	-0.68	0.736	0.707	5.35E-171
HNRNPM	2.62E-211	-0.67	0.78	0.723	5.97E-207
TK1	2.63E-145	-0.67	0.619	0.696	6.00E-141

BRIX1	1.03E-159	-0.67	0.722	0.691	2.36E-155
TPX2	4.87E-107	-0.67	0.526	0.66	1.11E-102
GMNN	1.15E-106	-0.66	0.377	0.635	2.64E-102
TFDP1	7.65E-138	-0.65	0.675	0.673	1.75E-133
SSRP1	3.70E-156	-0.64	0.725	0.689	8.44E-152
FEN1	1.56E-101	-0.63	0.385	0.645	3.56E-97
HNRNPU	1.17E-174	-0.63	0.779	0.728	2.66E-170
MCM4	3.40E-101	-0.62	0.283	0.61	7.76E-97
ALYREF	1.04E-182	-0.62	0.772	0.732	2.37E-178
TOP2A	2.10E-112	-0.62	0.146	0.535	4.80E-108
CBX3	2.29E-201	-0.61	0.779	0.737	5.22E-197
PPIH	2.33E-98	-0.61	0.639	0.667	5.32E-94
EBP	3.82E-104	-0.61	0.667	0.675	8.71E-100
NMU	1.26E-104	-0.61	0.557	0.674	2.88E-100
CCNA2	1.27E-143	-0.61	0.133	0.579	2.89E-139
NOP56	1.15E-161	-0.61	0.78	0.74	2.63E-157
HNRNPR	4.43E-150	-0.61	0.75	0.706	1.01E-145
KIF20B	2.08E-88	-0.60	0.33	0.603	4.74E-84
DUT	3.43E-173	-0.60	0.694	0.689	7.83E-169
SMC3	2.15E-104	-0.60	0.609	0.654	4.91E-100
EEF1E1	2.11E-117	-0.59	0.743	0.709	4.82E-113
TUBB4B	1.49E-131	-0.59	0.853	0.826	3.40E-127
HSPA5	4.69E-111	-0.59	0.777	0.758	1.07E-106
ORC6	3.29E-110	-0.59	0.251	0.6	7.51E-106
SMC4	1.49E-96	-0.59	0.362	0.632	3.40E-92
NOL11	8.04E-118	-0.58	0.696	0.677	1.83E-113
RAN	1.27E-186	-0.58	0.843	0.86	2.89E-182
ILF2	1.58E-167	-0.58	0.783	0.731	3.60E-163
CCNB2	3.32E-137	-0.58	0.181	0.611	7.58E-133
EIF4A1	1.05E-283	-0.57	0.873	0.847	2.39E-279
CENPN	2.30E-109	-0.57	0.492	0.664	5.24E-105
SPC25	5.04E-162	-0.57	0.08	0.543	1.15E-157
DNAJC9	1.78E-119	-0.57	0.597	0.657	4.06E-115
DBF4	4.53E-85	-0.57	0.454	0.609	1.03E-80
DEK	7.57E-178	-0.57	0.71	0.691	1.73E-173
MAGOHB	5.73E-82	-0.57	0.636	0.662	1.31E-77
FABP5	6.18E-152	-0.57	0.839	0.849	1.41E-147
EMG1	1.92E-120	-0.57	0.74	0.698	4.38E-116
CCT5	6.09E-159	-0.57	0.792	0.761	1.39E-154
MYBL2	5.04E-147	-0.57	0.156	0.601	1.15E-142
IER3	9.26E-87	-0.57	0.835	0.823	2.11E-82
SNRPE	2.00E-145	-0.56	0.785	0.77	4.58E-141
TOMM40	1.57E-156	-0.56	0.773	0.717	3.58E-152
NPM3	2.19E-118	-0.56	0.733	0.701	5.00E-114
RPA3	8.59E-122	-0.56	0.706	0.698	1.96E-117
CBX1	2.17E-110	-0.56	0.686	0.662	4.94E-106
TCP1	5.98E-148	-0.56	0.774	0.738	1.37E-143
HNRNPH3	2.04E-111	-0.56	0.712	0.686	4.66E-107

FAM92A	4.66E-88	-0.55	0.705	0.678	1.06E-83
SRRM1	1.62E-133	-0.55	0.772	0.729	3.70E-129
CSE1L	1.85E-98	-0.55	0.631	0.648	4.22E-94
PSMA3	2.10E-150	-0.55	0.781	0.759	4.80E-146
EXOSC8	6.26E-97	-0.55	0.515	0.66	1.43E-92
HELLS	8.02E-115	-0.55	0.278	0.614	1.83E-110
PLK1	7.21E-132	-0.55	0.13	0.559	1.65E-127
EIF4A3	2.68E-132	-0.55	0.758	0.719	6.11E-128
SLBP	1.29E-85	-0.55	0.719	0.684	2.94E-81
GTSE1	9.35E-119	-0.55	0.122	0.512	2.13E-114
SRSF4	3.64E-76	-0.54	0.666	0.653	8.31E-72
RBM8A	2.41E-97	-0.54	0.78	0.743	5.51E-93
SCD	2.96E-90	-0.54	0.537	0.632	6.76E-86
SNRNP40	3.17E-112	-0.54	0.703	0.676	7.23E-108
KIF23	3.80E-99	-0.54	0.206	0.555	8.67E-95
CDT1	6.44E-84	-0.53	0.38	0.615	1.47E-79
MRPS34	6.43E-117	-0.53	0.766	0.725	1.47E-112
ARL6IP1	1.89E-42	-0.53	0.751	0.706	4.31E-38
CEP55	1.14E-135	-0.53	0.129	0.559	2.60E-131
EXOSC9	5.29E-99	-0.53	0.591	0.65	1.21E-94
DDX39A	7.63E-89	-0.53	0.713	0.679	1.74E-84
SUPT16H	4.77E-122	-0.53	0.759	0.7	1.09E-117
H2AFV	1.97E-97	-0.53	0.737	0.7	4.49E-93
HSPA9	2.12E-129	-0.53	0.763	0.712	4.84E-125
UTP18	7.65E-90	-0.52	0.685	0.666	1.75E-85
NDC80	1.19E-116	-0.52	0.092	0.47	2.72E-112
SGO2	4.40E-79	-0.52	0.208	0.503	1.01E-74
NUDC	5.63E-131	-0.52	0.783	0.75	1.28E-126
CENPW	3.15E-94	-0.52	0.503	0.659	7.19E-90
MRPL11	1.81E-100	-0.52	0.753	0.714	4.14E-96
PNN	8.53E-105	-0.52	0.741	0.692	1.95E-100
MRTO4	3.54E-123	-0.52	0.758	0.704	8.08E-119
AURKB	1.88E-135	-0.52	0.135	0.568	4.29E-131
NOLC1	2.46E-88	-0.51	0.753	0.71	5.62E-84
LRR1	1.88E-68	-0.51	0.438	0.603	4.30E-64
KNSTRN	1.14E-60	-0.51	0.451	0.582	2.60E-56
HAT1	5.73E-93	-0.51	0.558	0.652	1.31E-88
MCM3	2.72E-115	-0.51	0.542	0.657	6.21E-111
C1QBP	3.26E-151	-0.51	0.821	0.823	7.44E-147
EIF5	1.68E-70	-0.50	0.766	0.711	3.83E-66
SRSF2	1.53E-170	-0.50	0.796	0.732	3.50E-166
NUCKS1	4.44E-85	-0.50	0.772	0.718	1.01E-80
DKC1	9.92E-110	-0.50	0.704	0.674	2.26E-105
HNRNPF	2.96E-121	-0.50	0.766	0.727	6.76E-117
BUB3	1.18E-60	-0.50	0.763	0.726	2.68E-56
MIS18A	2.37E-65	-0.50	0.311	0.551	5.40E-61
RFC4	2.17E-89	-0.50	0.303	0.599	4.95E-85
KPNB1	4.55E-111	-0.50	0.767	0.71	1.04E-106

TCOF1	3.82E-67	-0.49	0.51	0.632	8.72E-63
SFPQ	1.28E-111	-0.49	0.767	0.716	2.93E-107
GMPS	6.48E-84	-0.49	0.633	0.646	1.48E-79
SLIRP	5.24E-136	-0.49	0.806	0.783	1.20E-131
STEAP1	3.16E-92	-0.49	0.556	0.637	7.21E-88
C19orf48	7.23E-95	-0.49	0.646	0.674	1.65E-90
CCT2	4.31E-107	-0.49	0.794	0.772	9.84E-103
ERH	1.51E-108	-0.49	0.797	0.776	3.43E-104
CYCS	1.30E-121	-0.49	0.804	0.794	2.97E-117
CCDC58	9.18E-108	-0.49	0.707	0.67	2.09E-103
DNMT1	1.18E-117	-0.49	0.679	0.675	2.70E-113
HNRNPAB	1.64E-163	-0.49	0.803	0.722	3.75E-159
CDCA3	9.73E-122	-0.48	0.079	0.463	2.22E-117
PPID	3.20E-75	-0.48	0.702	0.671	7.31E-71
ITGB3BP	1.89E-96	-0.48	0.313	0.623	4.30E-92
MTHFD2	2.75E-99	-0.48	0.731	0.691	6.28E-95
PHF5A	8.78E-88	-0.48	0.672	0.648	2.00E-83
SERBP1	2.65E-147	-0.48	0.838	0.793	6.04E-143
SIVA1	1.50E-107	-0.48	0.772	0.726	3.42E-103
TOMM6	2.14E-100	-0.48	0.79	0.774	4.88E-96
NUP37	2.07E-61	-0.48	0.579	0.642	4.72E-57
HDGF	2.42E-87	-0.47	0.717	0.682	5.52E-83
PSMG1	1.67E-70	-0.47	0.702	0.679	3.80E-66
RPL7	1.12E-134	-0.47	0.902	0.919	2.56E-130
ANLN	2.00E-130	-0.47	0.118	0.538	4.56E-126
HMGB3	3.04E-78	-0.47	0.64	0.65	6.94E-74
BCLAF1	4.23E-75	-0.47	0.723	0.671	9.66E-71
XRCC5	4.07E-115	-0.47	0.786	0.738	9.28E-111
TIMM13	1.56E-116	-0.47	0.784	0.732	3.56E-112
SKA2	6.08E-86	-0.47	0.722	0.688	1.39E-81
HNRNPA3	1.03E-95	-0.47	0.788	0.74	2.34E-91
HNRNPC	2.49E-122	-0.47	0.799	0.755	5.67E-118
BAZ1B	2.52E-86	-0.47	0.693	0.653	5.74E-82
RRM1	9.93E-69	-0.47	0.392	0.603	2.27E-64
KIF22	1.40E-61	-0.47	0.449	0.6	3.20E-57
TOMM22	1.94E-82	-0.46	0.767	0.741	4.43E-78
TACC3	1.14E-81	-0.46	0.262	0.573	2.59E-77
GNL3	2.90E-117	-0.46	0.766	0.702	6.62E-113
LSM4	7.17E-90	-0.46	0.787	0.752	1.64E-85
GCSH	1.20E-102	-0.46	0.753	0.718	2.75E-98
EIF3A	3.09E-73	-0.46	0.787	0.76	7.05E-69
TCERG1	2.15E-68	-0.46	0.629	0.63	4.90E-64
EIF3E	2.65E-134	-0.46	0.805	0.772	6.05E-130
CHORDC1	5.27E-83	-0.46	0.738	0.687	1.20E-78
HNRNPDL	5.67E-103	-0.46	0.77	0.711	1.29E-98
BUB1	7.46E-138	-0.45	0.108	0.541	1.70E-133
PARP1	1.19E-100	-0.45	0.753	0.698	2.71E-96
HNRNPH1	3.46E-81	-0.45	0.751	0.7	7.90E-77

CDCA4	1.18E-74	-0.45	0.417	0.592	2.69E-70
CDCA5	1.07E-123	-0.45	0.103	0.505	2.44E-119
PRKDC	5.15E-82	-0.45	0.749	0.7	1.18E-77
ASPM	5.14E-117	-0.45	0.103	0.499	1.17E-112
RPL22L1	1.43E-130	-0.45	0.867	0.866	3.27E-126
LSM6	1.14E-67	-0.45	0.73	0.697	2.61E-63
MRPL1	1.42E-60	-0.44	0.717	0.671	3.25E-56
CCT6A	5.61E-94	-0.44	0.796	0.765	1.28E-89
PSIP1	6.45E-96	-0.44	0.632	0.644	1.47E-91
MCM10	2.33E-157	-0.44	0.063	0.516	5.31E-153
ID1	8.43E-28	-0.44	0.662	0.646	1.92E-23
CENPE	3.49E-68	-0.44	0.18	0.449	7.97E-64
CENPM	9.58E-92	-0.44	0.242	0.587	2.19E-87
RPS29	6.52E-152	-0.44	0.929	0.918	1.49E-147
DDX46	5.50E-93	-0.44	0.772	0.705	1.26E-88
AKAP12	4.92E-62	-0.44	0.82	0.801	1.12E-57
METAP2	1.20E-89	-0.44	0.77	0.716	2.73E-85
XPO1	3.00E-63	-0.43	0.69	0.665	6.85E-59
FBL	1.92E-131	-0.43	0.729	0.699	4.39E-127
UMPS	2.25E-49	-0.43	0.645	0.636	5.14E-45
TRIM28	5.06E-95	-0.43	0.784	0.722	1.15E-90
CLSPN	3.73E-120	-0.43	0.206	0.613	8.51E-116
POP5	2.84E-37	-0.43	0.707	0.67	6.47E-33
ITGAE	1.59E-48	-0.43	0.652	0.64	3.62E-44
CDK4	3.86E-67	-0.43	0.687	0.672	8.80E-63
CISD2	2.25E-59	-0.43	0.713	0.669	5.14E-55
HSPH1	9.65E-66	-0.43	0.698	0.666	2.20E-61
EIF4G2	4.77E-70	-0.42	0.771	0.726	1.09E-65
MAGOH	1.43E-61	-0.42	0.747	0.703	3.27E-57
APEX1	1.03E-73	-0.42	0.738	0.695	2.35E-69
MTAP	2.91E-56	-0.42	0.729	0.683	6.64E-52
CMC2	1.09E-54	-0.42	0.741	0.698	2.48E-50
ECT2	1.02E-51	-0.42	0.464	0.584	2.33E-47
SNRPA	1.34E-57	-0.42	0.667	0.652	3.06E-53
STIP1	8.87E-98	-0.42	0.776	0.71	2.02E-93
PSMC3IP	3.25E-109	-0.42	0.147	0.526	7.42E-105
H2AFX	2.70E-81	-0.42	0.651	0.666	6.16E-77
SMC2	1.66E-69	-0.42	0.296	0.571	3.78E-65
TTK	4.03E-119	-0.42	0.071	0.455	9.21E-115
NUF2	3.86E-132	-0.41	0.094	0.516	8.80E-128
GAL	2.14E-50	-0.41	0.759	0.743	4.89E-46
NCAPD3	7.11E-62	-0.41	0.335	0.562	1.62E-57
RFC5	1.78E-83	-0.41	0.29	0.587	4.06E-79
IPO5	2.17E-57	-0.41	0.603	0.624	4.96E-53
DHX9	1.79E-70	-0.41	0.69	0.659	4.08E-66
FDPS	3.52E-86	-0.41	0.759	0.704	8.04E-82
CENPX	2.95E-128	-0.41	0.825	0.771	6.74E-124
PSMA4	3.85E-63	-0.41	0.79	0.778	8.79E-59

ANP32A	8.96E-77	-0.41	0.759	0.697	2.04E-72
MND1	4.57E-119	-0.41	0.137	0.545	1.04E-114
POLR2H	2.19E-59	-0.41	0.766	0.735	4.99E-55
GLRX5	1.70E-58	-0.41	0.746	0.709	3.88E-54
UQCC3	2.74E-52	-0.41	0.731	0.687	6.26E-48
HPF1	1.52E-55	-0.40	0.591	0.613	3.47E-51
EIF5A	5.08E-156	-0.40	0.85	0.834	1.16E-151
SLC3A2	1.12E-78	-0.40	0.767	0.721	2.55E-74
ABCE1	3.47E-77	-0.40	0.741	0.692	7.93E-73
OR51B5	1.36E-44	-0.40	0.611	0.605	3.09E-40
CDC6	5.06E-105	-0.40	0.187	0.562	1.15E-100
PABPC4	2.43E-78	-0.40	0.746	0.69	5.55E-74
CDCA2	2.96E-106	-0.40	0.085	0.444	6.76E-102
SKA3	7.45E-129	-0.40	0.095	0.496	1.70E-124
TPRKB	1.95E-73	-0.40	0.722	0.673	4.44E-69
CCT4	5.95E-85	-0.40	0.785	0.743	1.36E-80
MRPL3	1.96E-75	-0.40	0.756	0.704	4.47E-71
FKBP4	1.03E-89	-0.40	0.774	0.72	2.34E-85
TROAP	4.44E-104	-0.40	0.201	0.586	1.01E-99
NBN	6.00E-44	-0.40	0.678	0.641	1.37E-39
MANF	8.98E-45	-0.40	0.736	0.702	2.05E-40
UHRF1	4.74E-82	-0.39	0.215	0.519	1.08E-77
ASF1B	1.57E-119	-0.39	0.096	0.494	3.58E-115
LDHB	3.84E-88	-0.39	0.838	0.83	8.76E-84
ACTG1	1.81E-71	-0.39	0.948	0.963	4.13E-67
HSP90B1	7.98E-87	-0.39	0.811	0.775	1.82E-82
ACAT2	2.10E-34	-0.39	0.604	0.621	4.80E-30
RPS17	7.02E-100	-0.39	0.855	0.873	1.60E-95
TSR1	6.48E-51	-0.39	0.698	0.65	1.48E-46
MZT1	1.66E-60	-0.39	0.559	0.611	3.79E-56
NEK2	2.35E-111	-0.39	0.051	0.4	5.36E-107
MCM5	9.41E-63	-0.39	0.423	0.607	2.15E-58
SUMO2	3.47E-79	-0.39	0.83	0.838	7.92E-75
DNAJC8	5.38E-80	-0.39	0.752	0.7	1.23E-75
SF3B1	1.17E-63	-0.39	0.744	0.683	2.67E-59
PRMT1	9.80E-97	-0.38	0.793	0.745	2.24E-92
EIF2S1	4.65E-59	-0.38	0.752	0.704	1.06E-54
PSMD1	6.15E-40	-0.38	0.746	0.699	1.40E-35
PFDN2	4.56E-70	-0.38	0.792	0.761	1.04E-65
YARS	3.33E-37	-0.38	0.659	0.635	7.61E-33
HNRNPK	9.52E-81	-0.38	0.785	0.735	2.17E-76
UBA2	1.25E-46	-0.38	0.665	0.634	2.86E-42
RSL24D1	5.22E-78	-0.38	0.763	0.71	1.19E-73
LSM2	2.68E-62	-0.38	0.738	0.697	6.11E-58
CCT8	1.30E-91	-0.38	0.799	0.762	2.96E-87
TRMT112	3.72E-59	-0.38	0.79	0.761	8.48E-55
PPM1G	1.05E-87	-0.38	0.779	0.724	2.39E-83
COPRS	2.16E-55	-0.38	0.727	0.679	4.94E-51

XRCC6	4.70E-97	-0.38	0.797	0.753	1.07E-92
HNRNPA0	8.29E-47	-0.38	0.722	0.666	1.89E-42
PFDN4	1.79E-66	-0.37	0.754	0.693	4.07E-62
NXT1	1.97E-46	-0.37	0.635	0.635	4.50E-42
MSH6	6.83E-56	-0.37	0.247	0.488	1.56E-51
SMC1A	2.79E-53	-0.37	0.516	0.605	6.38E-49
DSCC1	5.62E-91	-0.37	0.219	0.559	1.28E-86
COA1	2.48E-47	-0.37	0.705	0.66	5.65E-43
CHEK1	8.15E-46	-0.37	0.414	0.563	1.86E-41
WDR43	4.31E-52	-0.37	0.744	0.687	9.83E-48
YBX1	1.01E-78	-0.37	0.882	0.897	2.31E-74
ESCO2	1.52E-120	-0.37	0.082	0.479	3.46E-116
ENO1	2.92E-102	-0.36	0.868	0.863	6.67E-98
DANCR	1.93E-47	-0.36	0.673	0.663	4.40E-43
AHSA1	4.24E-65	-0.36	0.764	0.714	9.68E-61
CD3EAP	1.18E-32	-0.36	0.61	0.609	2.69E-28
TOMM5	5.73E-54	-0.36	0.791	0.771	1.31E-49
FAM83D	9.47E-60	-0.36	0.155	0.404	2.16E-55
NUDT21	1.03E-29	-0.36	0.683	0.631	2.35E-25
NAA15	1.46E-62	-0.36	0.687	0.643	3.34E-58
ERP29	2.07E-56	-0.36	0.758	0.698	4.73E-52
SNRPB2	5.34E-53	-0.36	0.764	0.718	1.22E-48
HMGN1	3.26E-79	-0.36	0.806	0.763	7.45E-75
RPL34	4.86E-90	-0.36	0.902	0.911	1.11E-85
NCAPH	1.15E-127	-0.36	0.067	0.462	2.63E-123
PRPF40A	5.11E-45	-0.36	0.765	0.714	1.17E-40
CMSS1	2.10E-56	-0.36	0.719	0.666	4.79E-52
KIF4A	4.23E-96	-0.36	0.121	0.462	9.66E-92
BUB1B	2.27E-109	-0.35	0.113	0.493	5.18E-105
EXOSC3	2.82E-55	-0.35	0.711	0.662	6.43E-51
PLP2	3.14E-37	-0.35	0.747	0.713	7.16E-33
PSMC6	1.53E-40	-0.35	0.727	0.679	3.48E-36
POLE3	3.89E-70	-0.35	0.686	0.654	8.87E-66
RPSA	2.09E-78	-0.35	0.913	0.938	4.78E-74
CYB5B	6.28E-41	-0.35	0.726	0.672	1.43E-36
DCAF13	4.53E-45	-0.35	0.741	0.696	1.03E-40
TIPIN	2.72E-58	-0.35	0.444	0.601	6.20E-54
KIF2C	7.74E-138	-0.35	0.065	0.484	1.77E-133
MAZ	4.68E-56	-0.35	0.705	0.669	1.07E-51
SUGT1	3.48E-52	-0.35	0.758	0.705	7.94E-48
SRPK1	1.36E-45	-0.35	0.746	0.682	3.09E-41
CHAF1A	2.28E-90	-0.35	0.215	0.554	5.21E-86
CCDC34	3.28E-34	-0.35	0.455	0.574	7.48E-30
THOC6	7.69E-50	-0.35	0.329	0.522	1.75E-45
HSPA1B	5.30E-46	-0.35	0.692	0.657	1.21E-41
SMARCA5	4.79E-51	-0.35	0.734	0.672	1.09E-46
TOP1	2.96E-74	-0.34	0.804	0.74	6.76E-70
PRDX3	2.58E-46	-0.34	0.764	0.731	5.90E-42

MELK	5.97E-75	-0.34	0.236	0.524	1.36E-70
ODC1	4.39E-39	-0.34	0.763	0.718	1.00E-34
IER2	3.32E-76	-0.34	0.772	0.701	7.57E-72
CCT3	1.10E-79	-0.34	0.82	0.803	2.51E-75
BZW1	2.07E-41	-0.34	0.763	0.713	4.73E-37
FBXO5	5.27E-85	-0.34	0.137	0.47	1.20E-80
NCAPD2	1.04E-72	-0.34	0.239	0.534	2.36E-68
BANF1	2.18E-46	-0.34	0.771	0.733	4.97E-42
DCTPP1	1.81E-70	-0.34	0.752	0.707	4.14E-66
POLR3K	1.20E-48	-0.34	0.642	0.63	2.74E-44
DHFR	5.85E-44	-0.34	0.356	0.565	1.34E-39
ACSL3	1.44E-46	-0.34	0.566	0.605	3.29E-42
RHEB	1.95E-27	-0.34	0.751	0.714	4.44E-23
CENPV	2.42E-54	-0.34	0.397	0.595	5.52E-50
GPN3	2.45E-41	-0.34	0.698	0.657	5.59E-37
TRIP13	6.25E-40	-0.33	0.403	0.54	1.43E-35
SRSF1	1.31E-54	-0.33	0.718	0.667	2.98E-50
DDX39B	1.17E-58	-0.33	0.744	0.686	2.67E-54
CEBPZ	1.14E-53	-0.33	0.682	0.643	2.60E-49
PSAT1	3.43E-33	-0.33	0.503	0.577	7.84E-29
LYAR	7.62E-56	-0.33	0.744	0.691	1.74E-51
TFAM	7.41E-52	-0.33	0.638	0.634	1.69E-47
PGP	1.70E-62	-0.33	0.734	0.684	3.89E-58
PRR11	1.28E-63	-0.33	0.169	0.436	2.92E-59
CHCHD3	1.14E-33	-0.33	0.698	0.658	2.60E-29
COMMD4	1.88E-67	-0.33	0.714	0.676	4.28E-63
LMNB1	1.96E-107	-0.33	0.125	0.504	4.47E-103
MGST1	1.50E-32	-0.33	0.741	0.709	3.42E-28
SHMT2	3.67E-54	-0.33	0.704	0.659	8.38E-50
LDHA	5.88E-80	-0.33	0.839	0.823	1.34E-75
PSMA1	1.00E-49	-0.33	0.786	0.746	2.29E-45
SRSF10	2.17E-59	-0.33	0.744	0.683	4.94E-55
TMEM258	4.03E-32	-0.33	0.772	0.732	9.19E-28
EPCAM	8.92E-22	-0.33	0.766	0.757	2.03E-17
MRPS2	5.91E-44	-0.33	0.725	0.671	1.35E-39
VRK1	1.18E-50	-0.33	0.478	0.603	2.69E-46
CCDC137	6.71E-60	-0.32	0.711	0.666	1.53E-55
RPL22	1.68E-95	-0.32	0.887	0.874	3.82E-91
PDAP1	5.43E-63	-0.32	0.774	0.721	1.24E-58
KRR1	5.73E-45	-0.32	0.749	0.693	1.31E-40
SNRPC	2.60E-34	-0.32	0.759	0.719	5.93E-30
YRDC	1.57E-32	-0.32	0.699	0.659	3.59E-28
GRWD1	4.82E-45	-0.32	0.637	0.634	1.10E-40
EEF1AKMT2	2.72E-47	-0.32	0.57	0.599	6.20E-43
MRPL13	2.84E-57	-0.32	0.763	0.716	6.49E-53
GLO1	1.10E-45	-0.32	0.746	0.707	2.50E-41
MPHOSPH6	3.83E-41	-0.32	0.717	0.665	8.75E-37
WDR34	1.49E-43	-0.32	0.611	0.637	3.40E-39

BTG3	1.13E-52	-0.32	0.733	0.678	2.59E-48
MCM6	3.10E-55	-0.32	0.371	0.58	7.07E-51
KIF14	1.12E-83	-0.32	0.045	0.332	2.55E-79
TARS	6.26E-40	-0.32	0.746	0.683	1.43E-35
SPCS2	9.37E-56	-0.32	0.757	0.703	2.14E-51
DNAJB11	2.16E-32	-0.32	0.711	0.669	4.93E-28
USP16	4.29E-36	-0.32	0.699	0.64	9.79E-32
NDUFAF4	2.49E-30	-0.32	0.698	0.662	5.68E-26
GART	5.47E-57	-0.32	0.704	0.647	1.25E-52
ACYP1	9.41E-49	-0.32	0.649	0.631	2.15E-44
RPL10A	5.08E-102	-0.32	0.904	0.897	1.16E-97
NIP7	2.34E-31	-0.32	0.716	0.665	5.35E-27
NSMCE4A	2.17E-49	-0.32	0.691	0.647	4.95E-45
SMC6	1.72E-33	-0.32	0.558	0.567	3.92E-29
DDX23	1.49E-46	-0.32	0.651	0.617	3.40E-42
PGD	2.91E-41	-0.32	0.694	0.652	6.65E-37
PHB2	2.77E-74	-0.32	0.791	0.735	6.31E-70
BOD1	4.27E-42	-0.32	0.605	0.595	9.74E-38
PNO1	7.19E-39	-0.32	0.694	0.656	1.64E-34
NR2F6	9.96E-37	-0.32	0.73	0.689	2.27E-32
POLR2C	1.94E-24	-0.32	0.699	0.629	4.44E-20
H1FX	8.31E-40	-0.32	0.632	0.628	1.90E-35
BCL2L12	4.64E-52	-0.31	0.61	0.642	1.06E-47
PCNA	3.01E-57	-0.31	0.761	0.714	6.86E-53
H3F3B	8.88E-59	-0.31	0.887	0.897	2.03E-54
LMNB2	3.88E-37	-0.31	0.578	0.588	8.85E-33
GAR1	1.61E-45	-0.31	0.691	0.651	3.67E-41
EIF1AX	3.21E-58	-0.31	0.777	0.721	7.32E-54
SGO1	1.28E-111	-0.31	0.074	0.447	2.93E-107
IDI1	1.35E-36	-0.31	0.706	0.65	3.08E-32
ARL6IP6	2.20E-37	-0.31	0.385	0.508	5.02E-33
SRRT	6.94E-38	-0.31	0.561	0.595	1.58E-33
EIF3J	6.71E-48	-0.31	0.738	0.682	1.53E-43
TMA7	2.75E-86	-0.31	0.873	0.838	6.27E-82
RBBP4	2.40E-37	-0.31	0.608	0.611	5.48E-33
G3BP1	2.05E-37	-0.31	0.744	0.682	4.67E-33
PIMREG	3.72E-104	-0.31	0.048	0.385	8.49E-100
PTMS	1.65E-55	-0.31	0.796	0.745	3.77E-51
PPIF	1.44E-34	-0.31	0.731	0.683	3.28E-30
EPRS	2.12E-25	-0.31	0.734	0.678	4.85E-21
HAUS1	3.22E-41	-0.31	0.515	0.591	7.35E-37
STOML2	2.29E-35	-0.31	0.769	0.73	5.23E-31
NUDT15	3.34E-43	-0.31	0.604	0.609	7.62E-39
HDAC2	8.88E-38	-0.31	0.734	0.674	2.03E-33
TEX30	1.36E-37	-0.31	0.51	0.572	3.09E-33
RFC2	3.56E-39	-0.31	0.491	0.601	8.11E-35
UTP11	8.48E-57	-0.31	0.718	0.667	1.93E-52
SNRPA1	6.01E-29	-0.31	0.751	0.704	1.37E-24

KHSRP	2.52E-41	-0.30	0.651	0.619	5.76E-37
NOP58	4.16E-60	-0.30	0.737	0.681	9.49E-56
GLOD4	1.56E-47	-0.30	0.667	0.639	3.55E-43
ABHD10	1.67E-27	-0.30	0.592	0.579	3.81E-23
RABGGTB	3.12E-61	-0.30	0.722	0.665	7.11E-57
RPS19BP1	2.29E-40	-0.30	0.779	0.725	5.23E-36
RAD51C	6.56E-53	-0.30	0.718	0.677	1.50E-48
CNIH1	2.02E-47	-0.30	0.716	0.667	4.60E-43
USP10	6.02E-22	-0.30	0.717	0.658	1.37E-17
TXNRD1	2.22E-34	-0.30	0.764	0.715	5.07E-30
HPRT1	4.59E-32	-0.30	0.656	0.631	1.05E-27
MRPL18	4.72E-27	-0.30	0.753	0.722	1.08E-22
NDUFB6	1.80E-42	-0.30	0.739	0.688	4.11E-38
MITD1	7.36E-52	-0.30	0.685	0.652	1.68E-47
EIF2S3	1.37E-44	-0.30	0.737	0.687	3.12E-40
KHDRBS1	3.33E-48	-0.30	0.745	0.679	7.59E-44
HAUS6	9.27E-41	-0.30	0.407	0.528	2.12E-36
NARS	8.66E-53	-0.30	0.751	0.68	1.98E-48
GTPBP4	1.94E-34	-0.30	0.743	0.686	4.42E-30
RPS3	8.01E-51	-0.30	0.94	0.963	1.83E-46
WDR75	6.12E-34	-0.30	0.658	0.628	1.40E-29
RPF1	8.52E-52	-0.30	0.684	0.658	1.94E-47
SRSF6	1.06E-29	-0.30	0.722	0.674	2.41E-25
SDF2L1	4.90E-71	-0.30	0.799	0.735	1.12E-66
RPL3	5.18E-79	-0.30	0.929	0.931	1.18E-74
WBP11	1.02E-51	-0.30	0.627	0.638	2.33E-47
EIF4E	9.94E-45	-0.30	0.745	0.688	2.27E-40
TMA16	3.18E-35	-0.30	0.685	0.641	7.25E-31
USP1	3.03E-46	-0.30	0.4	0.565	6.92E-42
SLC1A5	3.07E-54	-0.30	0.723	0.673	7.00E-50
RBX1	1.48E-30	-0.30	0.78	0.741	3.37E-26
RBBP8	6.01E-32	-0.30	0.439	0.516	1.37E-27
SLTM	5.36E-37	-0.29	0.745	0.679	1.22E-32
DDX18	1.46E-82	-0.29	0.779	0.704	3.34E-78
MRPS28	1.12E-30	-0.29	0.727	0.672	2.55E-26
FUS	2.03E-89	-0.29	0.784	0.704	4.63E-85
HDDC2	2.47E-20	-0.29	0.733	0.685	5.65E-16
NAMPT	1.12E-23	-0.29	0.723	0.668	2.55E-19
SYNCRIP	2.53E-55	-0.29	0.769	0.71	5.78E-51
EIF3B	3.43E-51	-0.29	0.763	0.705	7.84E-47
LTV1	7.29E-38	-0.29	0.683	0.645	1.66E-33
LSM7	4.96E-66	-0.29	0.82	0.748	1.13E-61
ETF1	1.86E-24	-0.29	0.738	0.68	4.24E-20
RBM25	2.35E-44	-0.29	0.765	0.702	5.37E-40
RBM28	7.04E-38	-0.29	0.657	0.621	1.61E-33
MT1X	3.54E-20	-0.29	0.736	0.715	8.08E-16
PSMC1	3.90E-57	-0.29	0.778	0.721	8.89E-53
MAK16	8.73E-39	-0.29	0.602	0.614	1.99E-34

SNHG6	3.22E-76	-0.29	0.828	0.78	7.35E-72
RPP30	1.46E-43	-0.29	0.659	0.634	3.34E-39
FANCA	3.28E-61	-0.29	0.229	0.509	7.48E-57
YWHAQ	1.05E-32	-0.29	0.772	0.728	2.40E-28
LRPPRC	1.64E-43	-0.29	0.694	0.655	3.74E-39
ACIN1	1.77E-33	-0.29	0.667	0.627	4.03E-29
SHCBP1	6.82E-109	-0.29	0.094	0.471	1.56E-104
CDC123	5.09E-34	-0.29	0.745	0.696	1.16E-29
SEM1	1.25E-38	-0.29	0.788	0.75	2.85E-34
TIMM17A	1.35E-28	-0.29	0.743	0.705	3.09E-24
DYNC1LI1	2.13E-28	-0.29	0.712	0.659	4.87E-24
NAP1L1	5.83E-68	-0.29	0.778	0.723	1.33E-63
DDX5	9.44E-54	-0.29	0.803	0.759	2.16E-49
RUVBL1	1.11E-37	-0.29	0.746	0.695	2.54E-33
MRPL12	5.36E-58	-0.29	0.79	0.743	1.22E-53
CALM2	1.41E-45	-0.29	0.839	0.808	3.22E-41
THRAP3	3.05E-52	-0.28	0.747	0.684	6.96E-48
DKK1	8.46E-07	-0.28	0.741	0.711	0.019
KIF11	4.29E-97	-0.28	0.09	0.438	9.80E-93
PHF19	1.40E-43	-0.28	0.467	0.623	3.19E-39
MRPL22	4.41E-38	-0.28	0.745	0.692	1.01E-33
TIMM10	5.36E-41	-0.28	0.698	0.664	1.22E-36
PRPF38B	6.33E-32	-0.28	0.679	0.625	1.45E-27
PPAT	2.77E-49	-0.28	0.642	0.621	6.31E-45
POP7	8.79E-36	-0.28	0.737	0.684	2.01E-31
HOXA9	4.25E-24	-0.28	0.673	0.621	9.69E-20
SPDL1	9.16E-44	-0.28	0.227	0.438	2.09E-39
CHRNA5	6.88E-31	-0.28	0.329	0.451	1.57E-26
ACTL6A	9.94E-29	-0.28	0.524	0.571	2.27E-24
RSRC1	4.40E-37	-0.28	0.622	0.592	1.01E-32
BMS1	1.36E-56	-0.28	0.73	0.672	3.11E-52
SNHG7	6.99E-46	-0.28	0.738	0.682	1.59E-41
TXNDC12	7.45E-31	-0.28	0.707	0.643	1.70E-26
RRP9	4.82E-44	-0.28	0.662	0.633	1.10E-39
MCMBP	1.59E-62	-0.28	0.565	0.629	3.63E-58
SSBP1	7.92E-41	-0.28	0.797	0.766	1.81E-36
SARNP	1.17E-41	-0.28	0.738	0.677	2.67E-37
BRCA1	5.12E-61	-0.28	0.187	0.47	1.17E-56
PNPT1	2.32E-33	-0.28	0.506	0.538	5.29E-29
EIF4EBP1	9.09E-41	-0.28	0.73	0.677	2.07E-36
RPL6	7.13E-59	-0.28	0.908	0.916	1.63E-54
ERGIC2	5.50E-21	-0.28	0.712	0.646	1.25E-16
SNHG3	1.05E-40	-0.28	0.716	0.653	2.39E-36
ARHGAP11A	7.73E-95	-0.28	0.129	0.484	1.76E-90
NUP155	5.44E-36	-0.28	0.389	0.494	1.24E-31
EREG	8.75E-18	-0.28	0.747	0.703	2.00E-13
DNAJC7	1.24E-32	-0.28	0.747	0.693	2.83E-28
FANCI	4.37E-76	-0.28	0.182	0.503	9.97E-72

RPL38	2.92E-90	-0.27	0.937	0.907	6.66E-86
PCBD1	3.88E-15	-0.27	0.756	0.72	8.86E-11
CENPK	2.70E-57	-0.27	0.23	0.487	6.16E-53
KIF18A	3.61E-40	-0.27	0.213	0.414	8.25E-36
RNASEH2B	7.05E-29	-0.27	0.559	0.568	1.61E-24
EIF1	3.56E-46	-0.27	0.875	0.893	8.12E-42
SRM	1.42E-29	-0.27	0.77	0.738	3.23E-25
G2E3	2.04E-24	-0.27	0.451	0.458	4.65E-20
EXOSC2	3.76E-39	-0.27	0.436	0.563	8.58E-35
PRIM1	6.11E-60	-0.27	0.231	0.506	1.39E-55
HSPA4	7.22E-27	-0.27	0.75	0.697	1.65E-22
RPF2	1.73E-36	-0.27	0.712	0.65	3.94E-32
DIAPH1	2.53E-32	-0.27	0.698	0.63	5.77E-28
CENPU	4.55E-64	-0.27	0.168	0.459	1.04E-59
CCDC59	3.06E-65	-0.27	0.734	0.678	6.99E-61
SFXN4	3.71E-26	-0.27	0.658	0.633	8.47E-22
METTL2A	4.20E-25	-0.27	0.649	0.608	9.58E-21
PSMA5	3.35E-40	-0.27	0.779	0.745	7.64E-36
PDP1	1.75E-53	-0.27	0.758	0.708	4.00E-49
PAGR1	7.71E-45	-0.27	0.469	0.57	1.76E-40
TNPO1	1.20E-34	-0.27	0.653	0.609	2.74E-30
SF1	1.97E-35	-0.27	0.749	0.682	4.50E-31
ATIC	1.16E-23	-0.27	0.677	0.637	2.65E-19
PMP22	3.07E-38	-0.27	0.604	0.601	7.01E-34
SRSF11	1.08E-29	-0.27	0.756	0.699	2.46E-25
ERI1	1.85E-32	-0.27	0.544	0.568	4.22E-28
PIM3	9.62E-30	-0.27	0.72	0.665	2.19E-25
PKMYT1	5.87E-67	-0.27	0.2	0.495	1.34E-62
RFC1	1.78E-42	-0.27	0.716	0.657	4.06E-38
SLC7A5	3.23E-31	-0.27	0.639	0.641	7.36E-27
RPL5	2.92E-58	-0.27	0.894	0.904	6.66E-54
NUP54	5.75E-40	-0.27	0.552	0.579	1.31E-35
MRPS30	4.07E-31	-0.27	0.723	0.67	9.30E-27
HSPA14.1	3.74E-31	-0.27	0.539	0.562	8.54E-27
NFIC	3.95E-32	-0.27	0.714	0.648	9.01E-28
RPS2	4.21E-50	-0.27	0.994	0.994	9.60E-46
MSH2	3.05E-58	-0.27	0.244	0.5	6.95E-54
PPP1CC	1.50E-37	-0.27	0.751	0.703	3.43E-33
DIAPH3	1.17E-53	-0.26	0.256	0.516	2.66E-49
POLR2E	3.79E-36	-0.26	0.766	0.712	8.64E-32
CCT7	2.19E-48	-0.26	0.787	0.751	5.01E-44
ESD	1.07E-31	-0.26	0.739	0.688	2.44E-27
POLR2D	3.20E-26	-0.26	0.608	0.548	7.30E-22
PARPBP	2.05E-57	-0.26	0.179	0.427	4.67E-53
PUM3	2.59E-33	-0.26	0.635	0.613	5.91E-29
GGH	1.30E-15	-0.26	0.622	0.617	2.97E-11
SLC25A19	1.65E-28	-0.26	0.49	0.548	3.76E-24
COPS3	8.01E-40	-0.26	0.72	0.668	1.83E-35

IPO7	2.59E-34	-0.26	0.684	0.629	5.92E-30
CWC22	1.99E-23	-0.26	0.59	0.548	4.54E-19
EXOSC7	6.48E-33	-0.26	0.71	0.661	1.48E-28
SAFB	1.07E-44	-0.26	0.638	0.627	2.43E-40
SAAL1	1.09E-35	-0.26	0.484	0.579	2.48E-31
ARGLU1	1.08E-24	-0.26	0.731	0.663	2.47E-20
MPHOSPH10	2.38E-38	-0.26	0.705	0.655	5.43E-34
CHD4	3.03E-39	-0.26	0.744	0.673	6.92E-35
RAD51AP1	2.65E-96	-0.26	0.121	0.483	6.05E-92
COQ3	1.76E-24	-0.26	0.416	0.503	4.03E-20
CDCA8	2.24E-98	-0.26	0.102	0.462	5.12E-94
TMEM70	5.02E-23	-0.26	0.734	0.687	1.14E-18
PRPF4	2.63E-37	-0.26	0.659	0.622	6.00E-33
RNF126	2.38E-38	-0.26	0.719	0.665	5.43E-34
DNAJC19	1.56E-19	-0.26	0.718	0.665	3.56E-15
RFC3	2.41E-104	-0.26	0.125	0.502	5.51E-100
RAD23A	1.23E-19	-0.26	0.746	0.691	2.81E-15
POLD2	7.90E-40	-0.26	0.749	0.692	1.80E-35
CALR	1.51E-33	-0.26	0.811	0.81	3.45E-29
PLPP2	5.64E-31	-0.26	0.724	0.678	1.29E-26
TMEM106C	1.25E-28	-0.26	0.698	0.658	2.85E-24
PRPS1	6.28E-25	-0.26	0.613	0.575	1.43E-20
OSTC	2.11E-20	-0.26	0.759	0.714	4.81E-16
CDCA7	6.68E-65	-0.26	0.188	0.456	1.53E-60
FOXM1	1.66E-95	-0.26	0.146	0.508	3.79E-91
HJURP	3.78E-97	-0.26	0.052	0.376	8.62E-93
RPS7	7.20E-38	-0.26	0.902	0.934	1.64E-33
FKBP5	2.10E-31	-0.26	0.277	0.45	4.79E-27
SLC25A5	1.76E-31	-0.26	0.784	0.772	4.02E-27
NUP93	5.29E-28	-0.26	0.592	0.591	1.21E-23
CENPQ	1.97E-78	-0.26	0.125	0.44	4.50E-74
MRPL39	5.95E-25	-0.26	0.639	0.602	1.36E-20
RSL1D1	2.98E-34	-0.26	0.776	0.729	6.81E-30
SAPCD2	1.61E-29	-0.26	0.343	0.483	3.68E-25
DENR	1.50E-36	-0.26	0.704	0.646	3.42E-32
CENPA	6.00E-90	-0.26	0.13	0.479	1.37E-85
EMC2	1.35E-19	-0.26	0.629	0.599	3.08E-15
DHX15	1.27E-42	-0.26	0.664	0.629	2.90E-38
CDCA7L	3.52E-31	-0.26	0.404	0.507	8.04E-27
FIP1L1	1.84E-22	-0.25	0.679	0.624	4.20E-18
MRPL20	1.11E-16	-0.25	0.761	0.734	2.54E-12
CTCF	4.67E-26	-0.25	0.613	0.572	1.06E-21
PDXP	1.16E-25	-0.25	0.388	0.47	2.65E-21
CTDNEP1	5.55E-28	-0.25	0.718	0.663	1.27E-23
MRPL49	8.53E-29	-0.25	0.618	0.57	1.95E-24
PPP1R14B	1.74E-66	-0.25	0.817	0.773	3.97E-62
UCHL5	1.57E-41	-0.25	0.651	0.638	3.58E-37
MRPL19	1.75E-29	-0.25	0.732	0.675	3.99E-25

PDIA6	6.05E-20	-0.25	0.741	0.706	1.38E-15
C1orf174	5.42E-40	-0.25	0.589	0.578	1.24E-35
IER3IP1	5.59E-29	-0.25	0.73	0.679	1.28E-24
RAC3	2.57E-39	-0.25	0.717	0.672	5.86E-35
YWHAH	4.05E-19	-0.25	0.757	0.711	9.24E-15
ZNF511	1.28E-28	-0.25	0.549	0.568	2.93E-24
BLMH	1.19E-22	-0.25	0.511	0.514	2.71E-18
DNTTIP2	2.48E-56	-0.25	0.74	0.672	5.65E-52

DEGs CD4+ T cells 72h

gene	p_val	avg_log2FC	pct.1	pct.2	p_val_adj
BTG1	1.41E-09	0.45	0.934	0.871	3.21E-05
HLA-E	7.03E-13	0.37	0.944	0.875	1.60E-08
WASHC3	1.04E-07	0.28	0.504	0.377	0.0024
FTL	6.71E-07	0.28	0.985	0.977	0.0153
AC004687.1	3.02E-51	-1.06	0.401	0.655	6.90E-47
HIST1H4C	1.23E-06	-0.64	0.521	0.568	0.0281
MT2A	4.41E-11	-0.50	0.842	0.921	1.01E-06
HMGA1	8.85E-10	-0.43	0.628	0.709	2.02E-05
MT1E	5.70E-07	-0.43	0.45	0.579	0.013
VMP1	4.11E-11	-0.42	0.637	0.714	9.37E-07
HSPE1	2.15E-12	-0.41	0.779	0.829	4.91E-08
ATP5ME	1.99E-18	-0.41	0.698	0.759	4.55E-14
SLIRP	6.48E-16	-0.37	0.621	0.68	1.48E-11
DKK1	8.00E-10	-0.37	0.06	0.159	1.83E-05
AKAP12	2.46E-18	-0.36	0.049	0.191	5.62E-14
ATP5MD	1.10E-19	-0.35	0.789	0.841	2.51E-15
HSPD1	1.35E-07	-0.34	0.722	0.786	0.0031
POLR2L	9.94E-12	-0.34	0.704	0.775	2.27E-07
SNRPF	6.61E-14	-0.32	0.688	0.73	1.51E-09
RPL37A	3.47E-28	-0.32	0.996	0.996	7.92E-24
RPL22L1	3.71E-07	-0.31	0.66	0.729	0.0085
NCL	1.52E-11	-0.30	0.808	0.809	3.46E-07
METAP2	2.82E-08	-0.30	0.589	0.65	0.0006
SNHG25	5.38E-07	-0.29	0.292	0.396	0.0123
NDUFA1	1.24E-09	-0.29	0.732	0.78	2.83E-05
TLN1	1.26E-06	-0.29	0.532	0.575	0.0288
RPS29	9.23E-19	-0.28	0.999	0.995	2.11E-14
PET100	4.31E-08	-0.28	0.606	0.671	0.001
TMA7	2.82E-15	-0.28	0.866	0.879	6.43E-11
TPM3	1.30E-12	-0.28	0.791	0.811	2.98E-08
UQCRQ	2.75E-11	-0.27	0.669	0.705	6.28E-07
NDUFB1	1.15E-08	-0.26	0.672	0.698	0.0003
MRPL52	1.86E-07	-0.26	0.634	0.677	0.0042
ATP5MPL	1.69E-11	-0.26	0.755	0.789	3.85E-07
ATP5MF	9.67E-12	-0.26	0.734	0.77	2.21E-07

DEGs CD8+ T cells 72h

gene	p_val	avg_log2FC	pct.1	pct.2	p_val_adj
SLA	2.29E-09	0.38	0.711	0.568	5.23E-05
TXNIP	3.99E-07	0.33	0.368	0.24	0.009
LIMD2	6.21E-09	0.29	0.869	0.778	0.0001
ITM2B	7.02E-08	0.28	0.895	0.822	0.0016
PSAT1	5.53E-14	0.27	0.383	0.202	1.26E-09
CCL5	8.07E-07	0.26	0.997	0.998	0.0184
PHGDH	9.73E-15	0.25	0.32	0.146	2.22E-10
IL5	2.31E-12	-0.93	0.456	0.546	5.27E-08
AC004687.1	7.26E-49	-0.79	0.497	0.755	1.66E-44
MTRNR2L8	1.04E-20	-0.63	0.227	0.368	2.37E-16
SLIRP	2.13E-25	-0.38	0.835	0.854	4.85E-21
RPS29	7.09E-55	-0.36	0.999	1	1.62E-50
NCL	6.59E-13	-0.36	0.938	0.955	1.50E-08
RPL37A	9.36E-40	-0.35	0.994	0.998	2.14E-35
AKAP12	3.18E-27	-0.32	0.051	0.22	7.26E-23
ATP5MD	3.41E-19	-0.32	0.923	0.956	7.78E-15
VMP1	2.10E-11	-0.32	0.807	0.834	4.80E-07
HSPE1	9.70E-12	-0.31	0.931	0.955	2.21E-07
RPL38	5.41E-26	-0.30	0.99	0.992	1.23E-21
RPL22L1	7.14E-10	-0.30	0.856	0.882	1.63E-05
MAT2A	2.70E-18	-0.30	0.606	0.644	6.15E-14
ATP5ME	2.30E-16	-0.29	0.883	0.901	5.26E-12
RPS21	1.52E-36	-0.28	0.997	1	3.46E-32
RPL36	3.75E-24	-0.28	0.991	0.995	8.57E-20
MT2A	1.04E-08	-0.28	0.953	0.978	0.0002
LINC01871	1.48E-08	-0.28	0.83	0.857	0.0003
HMGA1	1.52E-09	-0.28	0.724	0.795	3.46E-05
MRPL12	1.17E-10	-0.27	0.772	0.807	2.66E-06
SNHG6	2.83E-13	-0.27	0.917	0.934	6.46E-09
SNHG25	8.37E-15	-0.27	0.395	0.482	1.91E-10
MIR155HG	1.77E-08	-0.27	0.702	0.734	0.0004
FOS	6.32E-07	-0.27	0.205	0.272	0.0144
TPM3	2.23E-15	-0.27	0.937	0.931	5.09E-11
RPL37	3.02E-27	-0.27	0.996	1	6.90E-23
AP2B1	4.62E-13	-0.26	0.603	0.629	1.05E-08
METAP2	2.89E-17	-0.25	0.783	0.77	6.60E-13
COX17	1.89E-11	-0.25	0.888	0.909	4.31E-07

Supplementary data 2

Pathway enrichment analysis of 1150 DEGs, "MSigDB Hallmark 2020" database

**Term**	**p-value**	**q-value**
E2F Targets	6.12E-83	3.00E-81
Myc Targets V1	2.85E-80	6.99E-79
G2-M Checkpoint	2.55E-53	4.16E-52
mTORC1 Signaling	1.37E-25	1.68E-24
p53 Pathway	3.72E-19	3.64E-18
DNA Repair	4.38E-15	3.58E-14
Unfolded Protein Response	5.41E-12	3.31E-11
Apoptosis	5.88E-10	3.20E-09
TNF-alpha Signaling via NF-kB	9.02E-10	4.42E-09
Mitotic Spindle	2.91E-09	1.30E-08
Cholesterol Homeostasis	2.44E-08	9.24E-08
UV Response Up	2.45E-08	9.24E-08
Oxidative Phosphorylation	1.37E-07	4.81E-07
Interferon Alpha Response	5.20E-07	1.70E-06
Interferon Gamma Response	1.37E-06	4.19E-06
Estrogen Response Late	1.17E-05	3.17E-05
Glycolysis	1.17E-05	3.17E-05
Androgen Response	1.42E-05	3.66E-05
Hypoxia	0.0002	0.0005
Epithelial Mesenchymal Transition	0.0002	0.0005
Fatty Acid Metabolism	0.0008	0.0017
Complement	0.0027	0.0057
Estrogen Response Early	0.0057	0.0114
Coagulation	0.0058	0.0114
Peroxisome	0.0066	0.0125
Apical Junction	0.0114	0.02
heme Metabolism	0.0114	0.02
PI3K/AKT/mTOR Signaling	0.0174	0.0294
Allograft Rejection	0.022	0.0359

Supplementary data 3

Cluster	Term	Overlap	P.value	Adjusted.P.value	Odds.Ratio	Combined.Score	Genes
1	neutrophil degranulation (GO:0043312)	30/481	1.53E-12	1.22E-09	5.5	150.4	CD63;CSTB;GRN;PTAFR;CST3;LGALS3;GM2A;FTH1;PSAP;CD59;CAPN1;ACAA1;ATP6V1D;SNAP29;S100A11;HMOX2;CTSB;SERPINB1;GSN;TRAPPC1;ANXA2;HLA-B;CYBA;VAMP8;ERP44;SLPI;CSNK2B;CYSTM1;MAN2B1;YPEL5
1	neutrophil activation involved in immune response (GO:0002283)	30/485	1.89E-12	1.22E-09	5.5	147.9	CD63;CSTB;GRN;PTAFR;CST3;LGALS3;GM2A;FTH1;PSAP;CD59;CAPN1;ACAA1;ATP6V1D;SNAP29;S100A11;HMOX2;CTSB;SERPINB1;GSN;TRAPPC1;ANXA2;HLA-B;CYBA;VAMP8;ERP44;SLPI;CSNK2B;CYSTM1;MAN2B1;YPEL5
1	neutrophil mediated immunity (GO:0002446)	30/488	2.21E-12	1.22E-09	5.4	146.1	CD63;CSTB;GRN;PTAFR;CST3;LGALS3;GM2A;FTH1;PSAP;CD59;CAPN1;ACAA1;ATP6V1D;SNAP29;S100A11;HMOX2;CTSB;SERPINB1;GSN;TRAPPC1;ANXA2;HLA-B;CYBA;VAMP8;ERP44;SLPI;CSNK2B;CYSTM1;MAN2B1;YPEL5
1	autophagosome maturation (GO:0097352)	6/34	4.72E-06	1.96E-03	16.5	202.3	VAMP8;GABARAPL2;MAP1LC3B;TSG101;WIPI2;SNAP29
1	DNA damage response, signal transduction by p53 class mediator (GO:0030330)	7/74	5.20E-05	1.73E-02	8.1	79.5	PLK3;BTG2;CDKN1A;SP100;RPS27L;BAX;SFN
1	protein-containing complex disassembly (GO:0032984)	6/56	8.95E-05	2.48E-02	9.2	86.0	VAMP8;GABARAPL2;MAP1LC3B;TSG101;WIPI2;SNAP29
1	COPII vesicle coating (GO:0048208)	6/63	1.74E-04	2.96E-02	8.1	70.1	TRAPPC3;TRAPPC1;PDCD6;TRAPPC5;CD59;SEC22B
1	vesicle coating (GO:0006901)	6/63	1.74E-04	2.96E-02	8.1	70.1	TRAPPC3;TRAPPC1;PDCD6;TRAPPC5;CD59;SEC22B
1	vesicle targeting, rough ER to cis-Golgi (GO:0048207)	6/63	1.74E-04	2.96E-02	8.1	70.1	TRAPPC3;TRAPPC1;PDCD6;TRAPPC5;CD59;SEC22B
1	macroautophagy (GO:0016236)	8/120	1.85E-04	2.96E-02	5.5	47.4	VAMP8;GABARAPL2;MAP1LC3B;TSG101;CSNK2B;WIPI2;SNAP29;VPS28
1	mitotic G1 DNA damage checkpoint signaling [illegible]	6/65	2.06E-04	2.96E-02	7.8	66.3	PLK3;BTG2;CDKN1A;RPS27L;BAX;SFN
1	cellular response to starvation (GO:0009267)	9/158	2.44E-04	2.96E-02	4.7	38.9	GABARAPL2;CDKN1A;MAP1LC3B;ATP6V0E1;FAS;WIPI2;WDR45;ATP6V1D;ATP6V1F
1	low-density lipoprotein particle receptor catabolic process (GO:0032802)	3/10	2.49E-04	2.96E-02	32.6	270.9	AP2S1;CLTA;AP2B1
1	low-density lipoprotein receptor particle metabolic process (GO:0032799)	3/10	2.49E-04	2.96E-02	32.6	270.9	AP2S1;CLTA;AP2B1
1	receptor catabolic process (GO:0032801)	4/25	2.93E-04	3.04E-02	14.6	118.4	AP2S1;CLTA;AP2B1;LGMN
1	COPII-coated vesicle budding (GO:0090114)	6/70	3.10E-04	3.04E-02	7.2	58.2	TRAPPC3;TRAPPC1;PDCD6;TRAPPC5;CD59;SEC22B
1	wound healing (GO:0042060)	6/70	3.10E-04	3.04E-02	7.2	58.2	AHNAK2;MYH9;PPL;TSKU;CORO1B;PLEC
1	clathrin-dependent endocytosis (GO:0072583)	4/26	3.43E-04	3.17E-02	13.9	110.9	CLTB;AP2S1;CLTA;AP2B1
1	epithelial cell differentiation (GO:0030855)	7/101	3.69E-04	3.23E-02	5.7	45.4	LGALS3;GSTK1;ACADVL;TST;ANXA4;EDF1;CTSB

2	mRNA splicing, via spliceosome (GO:0000398)	20/274	9.83E-17	9.02E-14	16.3	599.7 SRRM2;RBM17;DDX1;ALYREF;LSM5;SRRM1;HNRNPM;PTBP1;SNRPD1;HNRNPA2B1;SNRPG;SRSF2;HNRNPD;SRSF3;SNRPB2;SNRPF;HNRNPH3;POLR2H;HNRNPA1;SNRPB
2	RNA splicing, via transesterification reactions with bulged adenosine as nucleophile (GO:0000377)	19/251	3.13E-16	1.44E-13	16.8	598.5 SRRM2;RBM17;ALYREF;LSM5;SRRM1;HNRNPM;PTBP1;SNRPD1;HNRNPA2B1;SNRPG;SRSF2;HNRNPD;SRSF3;SNRPB2;SNRPF;HNRNPH3;POLR2H;HNRNPA1;SNRPB
2	mRNA processing (GO:0006397)	19/300	8.32E-15	2.55E-12	13.8	447.7 SRRM2;RBM17;ALYREF;LSM5;SRRM1;HNRNPM;PTBP1;SNRPD1;HNRNPA2B1;SNRPG;SRSF2;HNRNPD;SRSF3;SNRPB2;SNRPF;HNRNPH3;POLR2H;HNRNPA1;SNRPB
2	DNA metabolic process (GO:0006259)	18/277	2.88E-14	6.62E-12	14.1	438.4 RFC5;CDT1;DNMT1;FEN1;NPM1;MCM7;XRCC5;PTGES3;GMNN;NUDT1;CDC6;HMGB1;ORC6;TRIM28;ORC3;MCM3;MCM4;RAN
2	DNA replication (GO:0006260)	12/108	1.28E-12	2.34E-10	24.0	657.7 RFC5;FEN1;ORC6;MCM7;ORC3;PCLAF;MCM3;MCMBP;MCM4;CLSPN;CDC6;PTMS
2	protein-DNA complex assembly (GO:0065004)	12/143	3.68E-11	5.18E-09	17.6	422.1 CDT1;NPM1;ORC6;CHAF1A;MCM7;ORC3;GMNN;MCM3;MCM4;CDC6;ANP32B;RBX1
2	DNA replication initiation (GO:0006270)	8/38	3.95E-11	5.18E-09	49.5	1185.3 CDT1;ORC6;MCM7;ORC3;GMNN;MCM3;MCM4;CDC6
2	DNA-dependent DNA replication (GO:0006261)	11/129	2.10E-10	2.41E-08	17.7	394.8 RFC5;CDT1;GINS2;ORC6;MCM7;ORC3;GMNN;MCM3;MCMBP;MCM4;CDC6
2	nucleic acid metabolic process (GO:0090304)	9/71	2.76E-10	2.82E-08	27.1	597.5 PTBP1;HNRNPM;FEN1;DDX1;HNRNPA2B1;HNRNPD;POLR2H;HNRNPA1;RAN
2	histone exchange (GO:0043486)	7/38	1.88E-09	1.73E-07	41.5	834.0 NPM1;CENPW;NASP;ANP32E;CENPM;CENPN;ITGB3BP
2	pre-replicative complex assembly (GO:0036388)	8/64	3.16E-09	2.64E-07	26.5	518.2 CDT1;ORC6;MCM7;ORC3;GMNN;MCM3;MCM4;CDC6
2	RNA processing (GO:0006396)	11/179	6.89E-09	5.27E-07	12.4	233.3 PTBP1;SNRPD1;HNRNPA2B1;SNRPG;SRSF2;HNRNPD;SNRPF;HNRNPH3;LSM5;SRRM1;SNRPB
2	CENP-A containing chromatin organization (GO:0061641)	6/30	1.68E-08	1.10E-06	45.6	815.5 NPM1;CENPW;NASP;CENPM;CENPN;ITGB3BP
2	CENP-A containing nucleosome assembly (GO:0034080)	6/30	1.68E-08	1.10E-06	45.6	815.5 NPM1;CENPW;NASP;CENPM;CENPN;ITGB3BP
2	chromatin remodeling at centromere (GO:0031055)	6/32	2.54E-08	1.56E-06	42.0	735.3 NPM1;CENPW;NASP;CENPM;CENPN;ITGB3BP
2	cellular macromolecule biosynthetic process (GO:0034645)	13/314	3.20E-08	1.84E-06	8.3	143.1 RFC5;FEN1;MCM7;CDC6;PTMS;ORC6;ORC3;PCLAF;MCM3;MCM4;CLSPN;POLR2H;PTMA
2	centromere complex assembly (GO:0034508)	6/37	6.37E-08	3.44E-06	35.3	584.1 NPM1;CENPW;NASP;CENPM;CENPN;ITGB3BP
2	pre-replicative complex assembly involved in nuclear cell cycle DNA replication (GO:0006267)	4/8	7.13E-08	3.64E-06	179.1	2947.4 MCM7;ORC3;MCM3;MCM4
2	DNA replication-independent nucleosome assembly [illegible]	6/39	8.86E-08	4.28E-06	33.1	537.8 NPM1;CENPW;NASP;CENPM;CENPN;ITGB3BP
2	RNA splicing (GO:0008380)	8/98	9.56E-08	4.39E-06	16.4	265.8 PTBP1;SNRPD1;SNRPG;SRSF2;SNRPF;HNRNPH3;SRRM1;SNRPB
2	nucleocytoplasmic transport (GO:0006913)	6/40	1.04E-07	4.53E-06	32.1	516.8 NPM1;SNRPD1;SNRPG;SNRPF;HNRNPA1;SNRPB
2	nuclear DNA replication (GO:0033260)	4/11	3.32E-07	1.38E-05	102.3	1526.7 MCM7;ORC3;MCM3;MCM4
2	double-strand break repair via break-induced replication (GO:0000727)	4/12	4.95E-07	1.98E-05	89.5	1299.9 GINS2;MCM7;MCM3;MCM4
2	ribonucleoprotein complex assembly (GO:0022618)	8/136	1.19E-06	4.56E-05	11.5	157.4 HSP90AB1;SNRPD1;XRCC5;DDX1;PTGES3;SNRPG;SNRPF;SNRPB

2	mRNA metabolic process (GO:0016071)	7/95	1.26E-06	4.62E-05	14.6	198.1	PTBP1;HNRNPA2B1;SNRPG;SRSF2;SNRPF;LSM5;SNRPB
2	RNA export from nucleus (GO:0006405)	7/106	2.63E-06	9.30E-05	13.0	166.4	ALYREF;HNRNPA2B1;SRSF2;SRSF3;HNRNPA1;RAN;SRRM1
2	DNA strand elongation (GO:0022616)	4/18	2.98E-06	9.78E-05	51.1	650.7	GINS2;MCM7;MCM3;MCM4
2	DNA strand elongation involved in DNA replication [illegible]	4/18	2.98E-06	9.78E-05	51.1	650.7	GINS2;MCM7;MCM3;MCM4
2	RNA transport (GO:0050658)	6/76	4.99E-06	1.58E-04	15.6	190.2	ALYREF;HNRNPA2B1;SRSF2;SRSF3;HNRNPA1;SRRM1
2	import into nucleus (GO:0051170)	6/77	5.39E-06	1.65E-04	15.4	186.4	SNRPD1;SNRPG;SNRPF;HNRNPA1;RAN;SNRPB
2	nuclear export (GO:0051168)	6/84	8.94E-06	2.65E-04	14.0	162.5	ALYREF;SRSF2;SRSF3;HNRNPA1;RAN;SRRM1
2	RNA metabolic process (GO:0016070)	7/133	1.18E-05	3.40E-04	10.2	115.3	HNRNPM;PTBP1;HNRNPA2B1;HNRNPD;HNRNPH3;POLR2H;HNRNPA1
2	nucleosome organization (GO:0034728)	6/94	1.71E-05	4.76E-04	12.4	135.9	SUPT16H;NPM1;CHAF1A;NASP;ANP32E;ANP32B
2	positive regulation of DNA biosynthetic process [illegible]	5/61	2.65E-05	7.16E-04	16.1	169.6	RFC5;HSP90AB1;XRCC5;PTGES3;HNRNPA1
2	cellular response to DNA damage stimulus (GO:0006974)	10/350	3.47E-05	9.10E-04	5.5	56.2	RFC5;FEN1;NPM1;TRIM28;MCM7;CBX3;XRCC5;UBR5;NUDT1;PCLAF
2	gene expression (GO:0010467)	10/356	4.01E-05	1.02E-03	5.4	54.5	ALYREF;HNRNPA2B1;SRSF2;HNRNPD;SRSF3;HNRNPH3;POLR2H;PTMA;SRRM1;HSPD1
2	spliceosomal snRNP assembly (GO:0000387)	4/38	6.58E-05	1.63E-03	21.0	202.6	SNRPD1;SNRPG;SNRPF;SNRPB
2	transcription by RNA polymerase II (GO:0006366)	9/320	9.78E-05	2.36E-03	5.3	49.3	SUPT16H;TFDP1;SNRPG;TCEA1;SNRPF;POLR2H;DEK;SSRP1;SNRPB
2	DNA replication checkpoint signaling (GO:0000076)	3/17	1.19E-04	2.79E-03	38.0	343.6	CDT1;CDC6;CLSPN
2	regulation of DNA metabolic process (GO:0051052)	4/46	1.40E-04	3.15E-03	17.0	151.0	CDT1;XRCC5;ALYREF;HNRNPD
2	positive regulation of nucleocytoplasmic transport [illegible]	4/46	1.40E-04	3.15E-03	17.0	151.0	TRIM28;UBR5;ANP32B;RAN
2	establishment of protein localization to mitochondrion [illegible]	4/48	1.66E-04	3.63E-03	16.2	141.4	TOMM40;TIMM13;HSPD1;TOMM22
2	response to unfolded protein (GO:0006986)	4/49	1.80E-04	3.84E-03	15.9	137.0	HSPA9;HSP90AB1;HSPE1;HSPD1
2	DNA duplex unwinding (GO:0032508)	4/51	2.10E-04	4.39E-03	15.2	128.8	MCM7;DDX1;MCM4;RBX1
2	positive regulation of transferase activity (GO:0051347)	6/148	2.15E-04	4.39E-03	7.7	64.6	RFC5;HSP90AB1;XRCC5;PTGES3;HNRNPA2B1;HNRNPD
2	double-strand break repair via homologous recombination (GO:0000724)	5/97	2.44E-04	4.77E-03	9.8	81.4	GINS2;FEN1;MCM7;MCM3;MCM4
2	regulation of DNA replication (GO:0006275)	4/53	2.44E-04	4.77E-03	14.6	121.3	CDT1;ORC3;GMNN;CDC6
2	regulation of chromosome organization (GO:0033044)	3/22	2.63E-04	4.92E-03	28.0	230.8	CDT1;XRCC5;HNRNPD
2	chaperone-mediated protein complex assembly [illegible]	3/22	2.63E-04	4.92E-03	28.0	230.8	HSP90AB1;PTGES3;HSPD1
2	mRNA-containing ribonucleoprotein complex export from nucleus (GO:0071427)	5/99	2.68E-04	4.92E-03	9.6	78.7	ALYREF;HNRNPA2B1;SRSF2;SRSF3;SRRM1
2	protein targeting to mitochondrion (GO:0006626)	4/55	2.82E-04	5.08E-03	14.0	114.5	TOMM40;TIMM13;TOMM22;HSPD1
2	transcription, DNA-templated (GO:0006351)	7/221	2.92E-04	5.15E-03	6.0	48.5	SUPT16H;TFDP1;TCEA1;POLR2H;DEK;SSRP1;PTMA
2	cytoskeleton-dependent intracellular transport [illegible]	3/23	3.02E-04	5.22E-03	26.6	215.7	TUBA1C;TUBA1B;TUBB
2	mRNA transport (GO:0051028)	5/104	3.37E-04	5.72E-03	9.1	72.6	ALYREF;HNRNPA2B1;SRSF2;SRSF3;SRRM1
2	nucleosome assembly (GO:0006334)	4/58	3.46E-04	5.78E-03	13.2	105.4	NPM1;CHAF1A;NASP;ANP32B

2	chromosome organization (GO:0051276)	5/106	3.68E-04	6.03E-03	8.9	70.4	CDT1;CENPW;HMGN2;RBX1;PHF19
2	mRNA export from nucleus (GO:0006406)	5/107	3.84E-04	6.18E-03	8.8	69.3	ALYREF;HNRNPA2B1;SRSF2;SRSF3;SRRM1
2	chaperone cofactor-dependent protein refolding [illegible]	3/26	4.37E-04	6.80E-03	23.1	178.9	HSPA9;PTGES3;HSPE1
2	DNA geometric change (GO:0032392)	3/26	4.37E-04	6.80E-03	23.1	178.9	DDX1;HNRNPA2B1;HMGB1
2	nuclear cell cycle DNA replication initiation (GO:1902315)	2/6	4.84E-04	7.17E-03	88.0	671.5	MCM3;MCM4
2	snoRNA localization (GO:0048254)	2/6	4.84E-04	7.17E-03	88.0	671.5	FBL;NOP58
2	mitotic DNA replication initiation (GO:1902975)	2/6	4.84E-04	7.17E-03	88.0	671.5	MCM3;MCM4
2	histone mRNA metabolic process (GO:0008334)	3/28	5.46E-04	7.84E-03	21.3	159.9	SNRPG;SNRPF;SNRPB
2	positive regulation of protein import into nucleus [illegible]	3/28	5.46E-04	7.84E-03	21.3	159.9	TRIM28;UBR5;RAN
2	transcription elongation from RNA polymerase II promoter (GO:0006368)	4/67	6.01E-04	8.49E-03	11.3	84.1	SUPT16H;TCEA1;POLR2H;SSRP1
2	positive regulation of protein import (GO:1904591)	3/30	6.71E-04	8.61E-03	19.7	143.9	TRIM28;UBR5;RAN
2	DNA-templated transcription, elongation (GO:0006354)	4/69	6.72E-04	8.61E-03	11.0	80.3	SUPT16H;TCEA1;POLR2H;SSRP1
2	RNA 3'-end processing (GO:0031123)	4/69	6.72E-04	8.61E-03	11.0	80.3	ALYREF;SRSF2;SRSF3;SRRM1
2	regulation of T cell mediated immune response to tumor cell (GO:0002840)	2/7	6.75E-04	8.61E-03	70.4	513.7	HMGB1;HSPD1
2	ribosomal large subunit export from nucleus (GO:0000055)	2/7	6.75E-04	8.61E-03	70.4	513.7	NPM1;RAN
2	ribosomal small subunit export from nucleus (GO:0000056)	2/7	6.75E-04	8.61E-03	70.4	513.7	NPM1;RAN
2	rRNA-containing ribonucleoprotein complex export from nucleus (GO:0071428)	2/7	6.75E-04	8.61E-03	70.4	513.7	NPM1;RAN
2	DNA-templated transcription, termination (GO:0006353)	4/70	7.10E-04	8.93E-03	10.8	78.5	SNRPG;SNRPF;POLR2H;SNRPB
2	'de novo' posttranslational protein folding (GO:0051084)	3/31	7.40E-04	9.18E-03	19.0	136.9	HSPA9;PTGES3;HSPE1
2	regulation of transcription involved in G1/S transition of mitotic cell cycle (GO:0000083)	3/32	8.13E-04	9.96E-03	18.3	130.5	CDT1;TFDP1;CDC6
2	transcription-coupled nucleotide-excision repair [illegible]	4/73	8.32E-04	1.00E-02	10.3	73.4	RFC5;TCEA1;POLR2H;RBX1
2	microtubule cytoskeleton organization involved in mitosis (GO:1902850)	5/128	8.69E-04	1.04E-02	7.3	51.5	CENPM;CENPN;ZWINT;ITGB3BP;RAN
2	regulation of endodeoxyribonuclease activity [illegible]	2/8	8.97E-04	1.06E-02	58.6	411.4	NPM1;HMGB1
2	nucleic acid phosphodiester bond hydrolysis (GO:0090305)	4/75	9.21E-04	1.06E-02	10.1	70.3	RFC5;FEN1;DDX1;RBX1
2	fibroblast growth factor receptor signaling pathway [illegible]	4/75	9.21E-04	1.06E-02	10.1	70.3	PTBP1;HNRNPM;POLR2H;HNRNPA1
2	termination of RNA polymerase II transcription [illegible]	3/34	9.73E-04	1.09E-02	17.2	119.0	SNRPG;SNRPF;SNRPB
2	positive regulation of telomerase activity (GO:0051973)	3/34	9.73E-04	1.09E-02	17.2	119.0	HSP90AB1;XRCC5;PTGES3
2	mRNA 3'-end processing (GO:0031124)	4/79	1.12E-03	1.23E-02	9.5	64.7	ALYREF;SRSF2;SRSF3;SRRM1
2	positive regulation of chromatin binding (GO:0035563)	2/9	1.15E-03	1.23E-02	50.3	340.2	CDT1;GMNN
2	regulation of protein import into nucleus (GO:0042306)	3/36	1.15E-03	1.23E-02	16.1	109.0	TRIM28;UBR5;RAN

2	positive regulation of telomere maintenance via telomere lengthening (GO:1904358)	3/36	1.15E-03	1.23E-02	16.1	109.0	XRCC5;HNRNPA2B1;HNRNPA1
2	regulation of gene expression, epigenetic (GO:0040029)	4/82	1.28E-03	1.36E-02	9.2	60.9	DNMT1;POLR2H;DEK;PHF19
2	chromatin organization (GO:0006325)	5/142	1.38E-03	1.43E-02	6.6	43.1	NPM1;CBX3;HMGN2;NPM3;PHF19
2	DNA replication-dependent nucleosome assembly [illegible]	2/10	1.43E-03	1.43E-02	44.0	288.0	CHAF1A;NASP
2	DNA replication-dependent nucleosome organization [illegible]	2/10	1.43E-03	1.43E-02	44.0	288.0	CHAF1A;NASP
2	mitotic DNA replication (GO:1902969)	2/10	1.43E-03	1.43E-02	44.0	288.0	MCM3;MCM4
2	mitotic DNA replication checkpoint signaling (GO:0033314)	2/10	1.43E-03	1.43E-02	44.0	288.0	CDC6;CLSPN
2	DNA integrity checkpoint signaling (GO:0031570)	3/39	1.46E-03	1.43E-02	14.8	96.5	CDT1;CLSPN;CDC6
2	G1/S transition of mitotic cell cycle (GO:0000082)	4/85	1.47E-03	1.43E-02	8.8	57.5	CDT1;TFDP1;NASP;CDC6
2	positive regulation of intracellular protein transport [illegible]	5/148	1.66E-03	1.60E-02	6.3	40.2	TFDP1;TRIM28;UBR5;ANP32B;RAN
2	DNA repair (GO:0006281)	7/298	1.69E-03	1.62E-02	4.4	27.9	RFC5;FEN1;NPM1;TRIM28;XRCC5;DDX1;NUDT1
2	positive regulation of DNA methylation-dependent heterochromatin assembly (GO:0090309)	2/11	1.74E-03	1.65E-02	39.1	248.3	DNMT1;TRIM28
2	regulation of mRNA splicing, via spliceosome (GO:0048024)	4/90	1.81E-03	1.68E-02	8.3	52.4	PTBP1;NCL;SRSF3;HNRNPA1
2	positive regulation of binding (GO:0051099)	4/90	1.81E-03	1.68E-02	8.3	52.4	CDT1;GMNN;HMGB1;RAN
2	cellular response to fibroblast growth factor stimulus [illegible]	4/92	1.96E-03	1.80E-02	8.1	50.5	PTBP1;HNRNPM;POLR2H;HNRNPA1
2	regulation of chromatin binding (GO:0035561)	2/12	2.08E-03	1.86E-02	35.2	217.2	CDT1;GMNN
2	regulation of DNA-dependent DNA replication [illegible]	2/12	2.08E-03	1.86E-02	35.2	217.2	CDT1;GMNN
2	chromatin assembly or disassembly (GO:0006333)	2/12	2.08E-03	1.86E-02	35.2	217.2	SUPT16H;CHAF1A
2	mitotic spindle organization (GO:0007052)	5/157	2.15E-03	1.89E-02	5.9	36.3	CENPM;CENPN;ZWINT;ITGB3BP;RAN
2	regulation of DNA repair (GO:0006282)	3/45	2.20E-03	1.93E-02	12.7	77.4	TRIM28;UBR5;DEK
2	regulation of telomerase activity (GO:0051972)	3/46	2.35E-03	2.01E-02	12.4	74.8	HSP90AB1;XRCC5;PTGES3
2	spliceosomal complex assembly (GO:0000245)	3/46	2.35E-03	2.01E-02	12.4	74.8	SNRPD1;DDX1;SNRPG
2	DNA replication-independent nucleosome organization [illegible]	2/13	2.45E-03	2.01E-02	32.0	192.2	SUPT16H;NASP
2	positive regulation of heterochromatin assembly [illegible]	2/13	2.45E-03	2.01E-02	32.0	192.2	DNMT1;TRIM28
2	ribosomal subunit export from nucleus (GO:0000054)	2/13	2.45E-03	2.01E-02	32.0	192.2	NPM1;RAN
2	kinetochore organization (GO:0051383)	2/13	2.45E-03	2.01E-02	32.0	192.2	CDT1;CENPW
2	mitotic DNA integrity checkpoint signaling (GO:0044774)	2/13	2.45E-03	2.01E-02	32.0	192.2	CDC6;CLSPN
2	response to UV (GO:0009411)	4/99	2.57E-03	2.08E-02	7.5	44.8	FEN1;NPM1;PCLAF;EIF2S1
2	RNA catabolic process (GO:0006401)	3/49	2.81E-03	2.27E-02	11.6	67.8	FEN1;HNRNPD;LSM5
2	regulation of DNA methylation-dependent heterochromatin assembly (GO:0090308)	2/14	2.85E-03	2.28E-02	29.3	171.8	DNMT1;TRIM28
2	nucleotide-excision repair (GO:0006289)	4/105	3.17E-03	2.51E-02	7.1	40.6	RFC5;TCEA1;POLR2H;RBX1

2	mitochondrion organization (GO:0007005)	5/175	3.43E-03	2.69E-02	5.3	29.9	TOMM40;TIMM13;SLIRP;TOMM22;HSPD1
2	negative regulation of DNA replication (GO:0008156)	2/16	3.73E-03	2.85E-02	25.1	140.5	GMNN;CDC6
2	regulation of mitotic sister chromatid separation [illegible]	2/16	3.73E-03	2.85E-02	25.1	140.5	PTTG1;CDC6
2	DNA unwinding involved in DNA replication (GO:0006268)	2/16	3.73E-03	2.85E-02	25.1	140.5	MCM7;MCM4
2	mitochondrial transport (GO:0006839)	3/56	4.11E-03	3.12E-02	10.0	55.1	TOMM40;TIMM13;TOMM22
2	positive regulation of gene expression, epigenetic [illegible]	3/57	4.32E-03	3.25E-02	9.8	53.5	ATAD2;POLR2H;DEK
2	alternative mRNA splicing, via spliceosome (GO:0000380)	2/18	4.72E-03	3.52E-02	22.0	117.7	RBM17;HNRNPM
2	regulation of cellular response to stress (GO:0080135)	4/118	4.81E-03	3.56E-02	6.2	33.4	NPM1;HSP90AB1;PTGES3;FKBP4
2	positive regulation of cysteine-type endopeptidase activity involved in apoptotic process (GO:0043280)	4/119	4.96E-03	3.64E-02	6.2	32.9	ANP32B;HMGB1;HSPE1;HSPD1
2	ribosome biogenesis (GO:0042254)	5/192	5.07E-03	3.69E-02	4.8	25.3	FBL;NPM1;NOP58;EXOSC8;RAN
2	cytoplasmic translational initiation (GO:0002183)	2/19	5.26E-03	3.80E-02	20.7	108.6	EIF4A1;EIF3I
3	microtubule cytoskeleton organization involved in mitosis (GO:1902850)	12/128	3.72E-15	1.78E-12	41.9	1390.4	CDC20;CCNB1;CENPF;PLK1;NUSAP1;TACC3;BIRC5;BUB1;DLGAP5;AURKB;MAD2L1;SPC25
3	mitotic chromosome condensation (GO:0007076)	8/27	9.85E-15	1.78E-12	158.3	5103.8	CCNB1;CSNK2A1;PLK1;NUSAP1;CDK1;NCAPG;NCAPD2;NCAPH
3	chromosome condensation (GO:0030261)	9/45	1.00E-14	1.78E-12	95.7	3084.4	TOP2A;CCNB1;CSNK2A1;PLK1;NUSAP1;CDK1;NCAPG;NCAPD2;NCAPH
3	mitotic sister chromatid segregation (GO:0000070)	11/102	1.18E-14	1.78E-12	48.0	1538.9	CCNB1;CSNK2A1;PRC1;PLK1;NUSAP1;CDK1;NCAPG;NCAPD2;DLGAP5;CEP55;NCAPH
3	mitotic spindle organization (GO:0007052)	12/157	4.47E-14	5.39E-12	33.4	1027.6	CDC20;TPX2;CCNB1;CENPF;PRC1;PLK1;BIRC5;BUB1;DLGAP5;AURKB;MAD2L1;SPC25
3	regulation of mitotic cell cycle (GO:0007346)	10/178	1.47E-10	1.48E-08	23.1	522.4	CDC20;CCNB1;UBE2C;PLK1;CDK1;MKI67;KIF20B;DLGAP5;CKS1B;MAD2L1
3	regulation of mitotic cell cycle phase transition [illegible]	10/188	2.52E-10	2.17E-08	21.8	481.1	CDC20;ANLN;TPX2;CCNB1;CENPF;UBE2C;PLK1;CDK1;HMMR;MAD2L1
3	positive regulation of cell cycle process (GO:0090068)	8/101	7.53E-10	5.68E-08	32.2	676.6	CCNB1;CENPV;DBF4;NUSAP1;MAP3K20;KIF20B;AURKB;MAD2L1
3	regulation of cell cycle process (GO:0010564)	8/106	1.11E-09	7.44E-08	30.6	630.1	DBF4;CSNK2A1;PRC1;PLK1;MKI67;KIF20B;BUB1;AURKB
3	regulation of cell cycle (GO:0051726)	11/296	1.34E-09	8.10E-08	15.2	309.9	CCNB2;CCNB1;CENPF;CSNK2A1;PLK1;MAP3K20;BIRC5;KIF20B;DLGAP5;CKS1B;CDKN3
3	spindle assembly checkpoint signaling (GO:0071173)	5/21	4.37E-09	2.03E-07	111.2	2139.9	CDC20;CENPF;PLK1;BUB1;MAD2L1
3	mitotic spindle assembly checkpoint signaling [illegible]	5/21	4.37E-09	2.03E-07	111.2	2139.9	CDC20;CENPF;PLK1;BUB1;MAD2L1
3	mitotic spindle checkpoint signaling (GO:0071174)	5/21	4.37E-09	2.03E-07	111.2	2139.9	CDC20;CENPF;PLK1;BUB1;MAD2L1
3	negative regulation of mitotic metaphase/anaphase transition (GO:0045841)	5/21	5.65E-09	2.43E-07	104.6	1987.2	CDC20;CENPF;PLK1;BUB1;MAD2L1
3	anaphase-promoting complex-dependent catabolic process (GO:0031145)	7/84	6.49E-09	2.61E-07	33.4	630.4	CDC20;CCNB1;UBE2C;PLK1;CDK1;AURKB;MAD2L1
3	mitotic cell cycle phase transition (GO:0044772)	9/209	1.36E-08	5.11E-07	17.1	309.5	CCNA2;CCNB2;CCNB1;DBF4;UBE2C;PLK1;CDK1;TACC3;CDKN3
3	regulation of cell cycle phase transition (GO:1901987)	7/95	1.54E-08	5.47E-07	29.2	526.0	CDC20;CCNB1;DBF4;UBE2C;PLK1;CDK1;MAD2L1

3	mitotic nuclear membrane disassembly (GO:0007077)	4/12	3.81E-08	1.27E-06	174.8	2986.9	CCNB2;CCNB1;PLK1;CDK1
3	nuclear membrane disassembly (GO:0051081)	4/14	7.66E-08	2.43E-06	139.9	2291.4	CCNB2;CCNB1;PLK1;CDK1
3	regulation of chromosome segregation (GO:0051983)	4/18	2.32E-07	7.00E-06	99.9	1525.7	CSNK2A1;MKI67;BUB1;AURKB
3	regulation of ubiquitin protein ligase activity [illegible]	4/21	4.51E-07	1.29E-05	82.2	1201.7	CDC20;UBE2C;PLK1;MAD2L1
3	regulation of mitotic metaphase/anaphase transition [illegible]	4/26	1.11E-06	3.05E-05	63.5	870.9	CCNB1;UBE2C;PLK1;DLGAP5
3	cytoskeleton-dependent cytokinesis (GO:0061640)	5/72	2.67E-06	7.00E-05	26.5	339.8	ANLN;PLK1;NUSAP1;CEP55;AURKB
3	regulation of cyclin-dependent protein serine/threonine kinase activity (GO:0000079)	5/82	5.09E-06	1.27E-04	23.0	280.7	CCNA2;CCNB2;CCNB1;PLK1;CDKN3
3	regulation of cytokinesis (GO:0032465)	5/84	5.73E-06	1.27E-04	22.4	270.9	CENPV;PRC1;PLK1;KIF20B;AURKB
3	positive regulation of ubiquitin protein ligase activity [illegible]	3/12	5.82E-06	1.27E-04	114.5	1380.6	CDC20;UBE2C;PLK1
3	regulation of exit from mitosis (GO:0007096)	4/39	5.95E-06	1.27E-04	39.9	480.2	CDC20;ANLN;UBE2C;MAD2L1
3	regulation of cell cycle G2/M phase transition [illegible]	5/85	6.07E-06	1.27E-04	22.2	266.2	TPX2;CENPF;PLK1;CDK1;HMMR
3	regulation of G2/M transition of mitotic cell cycle [illegible]	6/149	6.12E-06	1.27E-04	15.1	181.3	TPX2;CCNB1;CENPF;PLK1;CDK1;HMMR
3	regulation of mitotic sister chromatid separation [illegible]	3/16	1.47E-05	2.91E-04	79.3	882.2	UBE2C;PLK1;TACC3
3	mitotic cytokinesis (GO:0000281)	4/49	1.50E-05	2.91E-04	31.0	344.7	ANLN;PLK1;NUSAP1;CEP55
3	mitotic nuclear membrane organization (GO:0101024)	4/51	1.76E-05	3.21E-04	29.7	325.2	CCNB2;CCNB1;CDK1;LBR
3	mitotic nuclear membrane reassembly (GO:0007084)	4/51	1.76E-05	3.21E-04	29.7	325.2	CCNB2;CCNB1;CDK1;LBR
3	regulation of cyclin-dependent protein kinase activity [illegible]	4/54	2.21E-05	3.81E-04	27.9	299.2	CCNA2;CCNB2;CCNB1;CDKN3
3	nuclear membrane reassembly (GO:0031468)	4/54	2.21E-05	3.81E-04	27.9	299.2	CCNB2;CCNB1;CDK1;LBR
3	regulation of mitotic nuclear division (GO:0007088)	4/57	2.74E-05	4.59E-04	26.3	276.6	CCNB1;NUSAP1;MKI67;KIF20B
3	positive regulation of mitotic cell cycle phase transition [illegible]	4/58	2.94E-05	4.79E-04	25.8	269.7	CCNB1;UBE2C;CDK1;DLGAP5
3	G2/M transition of mitotic cell cycle (GO:0000086)	5/130	4.77E-05	7.57E-04	14.2	140.8	CCNA2;CCNB2;CCNB1;PLK1;CDK1
3	cell cycle G2/M phase transition (GO:0044839)	5/131	4.95E-05	7.65E-04	14.0	139.2	CCNA2;CCNB2;CCNB1;PLK1;CDK1
3	proteasome-mediated ubiquitin-dependent protein catabolic process (GO:0043161)	7/321	5.33E-05	8.04E-04	8.1	79.7	CDC20;CCNB1;UBE2C;PLK1;CDK1;AURKB;MAD2L1
3	mitotic nuclear division (GO:0140014)	4/74	7.68E-05	1.13E-03	19.9	188.7	TPX2;UBE2C;PLK1;NUSAP1
3	establishment of mitotic spindle localization (GO:0040001)	3/29	9.32E-05	1.34E-03	39.6	367.6	PLK1;NUSAP1;MAD2L1
3	positive regulation of ubiquitin-protein transferase activity (GO:0051443)	3/31	1.14E-04	1.60E-03	36.8	333.9	CDC20;UBE2C;PLK1
3	protein phosphorylation (GO:0006468)	8/496	1.26E-04	1.72E-03	6.0	54.0	CCNA2;CSNK2A1;COPS2;PLK1;MAP3K20;CDK1;BIRC5;AURKB
3	sister chromatid segregation (GO:0000819)	3/34	1.51E-04	2.02E-03	33.2	292.2	TOP2A;PLK1;NUSAP1
3	regulation of mitotic spindle organization (GO:0060236)	3/35	1.65E-04	2.16E-03	32.2	280.3	TPX2;PLK1;TACC3
3	positive regulation of cytokinesis (GO:0032467)	3/37	1.95E-04	2.41E-03	30.3	258.7	CENPV;KIF20B;AURKB
3	centromere complex assembly (GO:0034508)	3/37	1.95E-04	2.41E-03	30.3	258.7	CENPV;CENPF;DLGAP5

3	DNA metabolic process (GO:0006259)	6/277	1.96E-04	2.41E-03	7.9	67.6	TOP2A;DBF4;UBE2T;HMGB2;CDK1;KPNA2
3	phosphorylation (GO:0016310)	7/400	2.09E-04	2.52E-03	6.4	54.6	CSNK2A1;COPS2;PLK1;MAP3K20;CDK1;BIRC5;AURKB
3	regulation of metaphase/anaphase transition of cell cycle (GO:1902099)	2/8	2.53E-04	2.88E-03	112.6	932.6	UBE2C;PLK1
3	mitotic spindle elongation (GO:0000022)	2/8	2.53E-04	2.88E-03	112.6	932.6	PRC1;AURKB
3	mitotic spindle midzone assembly (GO:0051256)	2/8	2.53E-04	2.88E-03	112.6	932.6	PRC1;AURKB
3	chromosome organization (GO:0051276)	4/106	3.08E-04	3.40E-03	13.6	110.3	TOP2A;CENPV;COPS2;HMGB2
3	kinetochore assembly (GO:0051382)	2/9	3.25E-04	3.40E-03	96.5	775.3	CENPF;DLGAP5
3	DNA topological change (GO:0006265)	2/9	3.25E-04	3.40E-03	96.5	775.3	TOP2A;HMGB2
3	positive regulation of cell division (GO:0051781)	3/44	3.27E-04	3.40E-03	25.1	201.5	CENPV;KIF20B;AURKB
3	mitotic spindle assembly (GO:0090307)	3/44	3.27E-04	3.40E-03	25.1	201.5	TPX2;PRC1;AURKB
3	regulation of protein serine/threonine kinase activity [illegible]	4/111	3.68E-04	3.69E-03	13.0	102.9	CCNA2;CCNB2;CCNB1;CDKN3
3	modification-dependent protein catabolic process [illegible]	5/201	3.68E-04	3.69E-03	9.0	71.1	CDC20;UBE2C;PLK1;AURKB;MAD2L1
3	protein localization to condensed chromosome [illegible]	2/12	5.92E-04	5.85E-03	67.6	502.1	CDK1;AURKB
3	kinetochore organization (GO:0051383)	2/13	6.98E-04	6.62E-03	61.4	446.3	CENPF;DLGAP5
3	regulation of mitotic cell cycle spindle assembly checkpoint (GO:0090266)	2/13	6.98E-04	6.62E-03	61.4	446.3	CCNB1;MAD2L1
3	protein K48-linked ubiquitination (GO:0070936)	3/57	7.02E-04	6.62E-03	19.0	138.3	UBE2C;UBE2T;UBE3A
3	protein localization to kinetochore (GO:0034501)	2/14	8.13E-04	7.54E-03	56.3	400.5	CDK1;AURKB
3	cell cycle G1/S phase transition (GO:0044843)	3/63	9.41E-04	8.60E-03	17.1	119.4	CCNA2;DBF4;CDKN3
3	regulation of stem cell proliferation (GO:0072091)	2/17	1.21E-03	1.06E-02	45.0	302.5	KDM1A;HMGB2
3	cellular response to gamma radiation (GO:0071480)	2/17	1.21E-03	1.06E-02	45.0	302.5	KDM1A;MAP3K20
3	protein localization to chromosome, centromeric region [illegible]	2/17	1.21E-03	1.06E-02	45.0	302.5	CDK1;AURKB
3	regulation of signal transduction by p53 class mediator [illegible]	4/156	1.32E-03	1.13E-02	9.1	60.6	TPX2;CSNK2A1;CDK1;AURKB
3	regulation of spindle organization (GO:0090224)	2/18	1.36E-03	1.14E-02	42.2	278.7	TPX2;TACC3
3	DNA conformation change (GO:0071103)	2/18	1.36E-03	1.14E-02	42.2	278.7	TOP2A;HMGB2
3	regulation of cell division (GO:0051302)	3/76	1.62E-03	1.34E-02	14.1	90.4	PRC1;PLK1;AURKB
3	positive regulation of G2/M transition of mitotic cell cycle (GO:0010971)	2/20	1.68E-03	1.35E-02	37.5	239.7	CCNB1;CDK1
3	regulation of nuclear division (GO:0051783)	2/20	1.68E-03	1.35E-02	37.5	239.7	MKI67;KIF20B
3	positive regulation of protein ubiquitination (GO:0031398)	3/82	2.01E-03	1.59E-02	13.0	80.7	KDM1A;PLK1;UBE3A
3	regulation of chromosome organization (GO:0033044)	2/22	2.03E-03	1.59E-02	33.8	209.3	CENPV;BUB1
3	positive regulation of cell cycle G2/M phase transition [illegible]	2/23	2.22E-03	1.67E-02	32.2	196.4	CCNB1;CDK1
3	mitotic cell cycle checkpoint signaling (GO:0007093)	2/23	2.22E-03	1.67E-02	32.2	196.4	BUB1;AURKB

3	regulation of neural precursor cell proliferation [illegible]	2/23	2.22E-03	1.67E-02	32.2	196.4	KDM1A;SHCBP1
3	establishment of mitotic spindle orientation (GO:0000132)	2/24	2.42E-03	1.76E-02	30.7	184.9	PLK1;MAD2L1
3	apoptotic nuclear changes (GO:0030262)	2/24	2.42E-03	1.76E-02	30.7	184.9	TOP2A;HMGB2
3	response to gamma radiation (GO:0010332)	2/24	2.42E-03	1.76E-02	30.7	184.9	KDM1A;MAP3K20
3	protein K11-linked ubiquitination (GO:0070979)	2/29	3.52E-03	2.53E-02	25.0	141.2	UBE2C;UBE2T
3	regulation of protein catabolic process (GO:0042176)	3/102	3.74E-03	2.64E-02	10.4	57.9	CSNK2A1;AZIN1;MAD2L1
3	establishment of spindle orientation (GO:0051294)	2/30	3.77E-03	2.64E-02	24.1	134.5	PLK1;MAD2L1
3	ubiquitin-dependent protein catabolic process [illegible]	5/354	4.47E-03	3.09E-02	5.0	27.1	CDC20;UBE2C;PLK1;AURKB;MAD2L1
3	mitotic G2 DNA damage checkpoint signaling [illegible]	2/33	4.55E-03	3.12E-02	21.8	117.4	PLK1;CDK1
3	positive regulation of mitotic nuclear division [illegible]	2/36	5.39E-03	3.65E-02	19.8	103.6	NUSAP1;DLGAP5
3	regulation of protein binding (GO:0043393)	3/118	5.62E-03	3.76E-02	8.9	46.2	KDM1A;PLK1;AURKB
3	regulation of apoptotic process (GO:0042981)	7/742	7.21E-03	4.67E-02	3.4	16.7	TOP2A;PLK1;MAP3K20;CDK1;BIRC5;RRP1B;MAD2L1
3	regulation of ubiquitin-dependent protein catabolic process (GO:2000058)	2/42	7.28E-03	4.67E-02	16.9	83.0	CSNK2A1;UBE3A
3	regulation of DNA recombination (GO:0000018)	2/42	7.28E-03	4.67E-02	16.9	83.0	KDM1A;KPNA2
3	negative regulation of protein catabolic process [illegible]	2/42	7.28E-03	4.67E-02	16.9	83.0	AZIN1;MAD2L1
5	SRP-dependent cotranslational protein targeting to membrane (GO:0006614)	15/90	2.79E-32	2.23E-30	796.2	57848.8	RPL10;RPL34;RPLP0;RPSA;RPS15;RPS14;RPS27;RPS19;RPS18;RPS29;RPL37A;RPL13;RPL38;RPS21;RPS23
5	cytoplasmic translation (GO:0002181)	15/93	4.76E-32	2.23E-30	765.5	55207.2	RPL10;RPL34;RPLP0;RPSA;RPS15;RPS14;RPS27;RPS19;RPS18;RPS29;RPL37A;RPL13;RPL38;RPS21;RPS23
5	cotranslational protein targeting to membrane (GO:0006613)	15/94	5.66E-32	2.23E-30	755.7	54374.4	RPL10;RPL34;RPLP0;RPSA;RPS15;RPS14;RPS27;RPS19;RPS18;RPS29;RPL37A;RPL13;RPL38;RPS21;RPS23
5	protein targeting to ER (GO:0045047)	15/103	2.48E-31	7.32E-30	678.1	47789.1	RPL10;RPL34;RPLP0;RPSA;RPS15;RPS14;RPS27;RPS19;RPS18;RPS29;RPL37A;RPL13;RPL38;RPS21;RPS23
5	nuclear-transcribed mRNA catabolic process, nonsense-mediated decay (GO:0000184)	15/113	1.10E-30	2.59E-29	608.6	41986.2	RPL10;RPL34;RPLP0;RPSA;RPS15;RPS14;RPS27;RPS19;RPS18;RPS29;RPL37A;RPL13;RPL38;RPS21;RPS23
5	peptide biosynthetic process (GO:0043043)	15/162	3.26E-28	6.42E-27	404.8	25616.6	RPL10;RPL34;RPLP0;RPSA;RPS15;RPS14;RPS27;RPS19;RPS18;RPS29;RPL37A;RPL13;RPL38;RPS21;RPS23
5	nuclear-transcribed mRNA catabolic process (GO:0000956)	15/171	7.60E-28	1.28E-26	381.2	23805.6	RPL10;RPL34;RPLP0;RPSA;RPS15;RPS14;RPS27;RPS19;RPS18;RPS29;RPL37A;RPL13;RPL38;RPS21;RPS23
5	translation (GO:0006412)	15/214	2.48E-26	3.66E-25	298.2	17582.0	RPL10;RPL34;RPLP0;RPSA;RPS15;RPS14;RPS27;RPS19;RPS18;RPS29;RPL37A;RPL13;RPL38;RPS21;RPS23
5	cellular macromolecule biosynthetic process (GO:0034645)	15/314	8.96E-24	1.18E-22	197.5	10479.4	RPL10;RPL34;RPLP0;RPSA;RPS15;RPS14;RPS27;RPS19;RPS18;RPS29;RPL37A;RPL13;RPL38;RPS21;RPS23
5	gene expression (GO:0010467)	15/356	6.08E-23	7.17E-22	172.8	8838.4	RPL10;RPL34;RPLP0;RPSA;RPS15;RPS14;RPS27;RPS19;RPS18;RPS29;RPL37A;RPL13;RPL38;RPS21;RPS23
5	cellular protein metabolic process (GO:0044267)	15/417	6.71E-22	7.20E-21	146.1	7123.0	RPL10;RPL34;RPLP0;RPSA;RPS15;RPS14;RPS27;RPS19;RPS18;RPS29;RPL37A;RPL13;RPL38;RPS21;RPS23

5	rRNA metabolic process (GO:0016072)	10/162	1.58E-16	1.55E-15	130.4	4746.1	RPS15;RPS14;RPS27;RPL10;RPS19;RPL34;RPLP0;RPL37A;RPL13;RPL38
5	rRNA processing (GO:0006364)	10/173	3.09E-16	2.81E-15	121.6	4341.9	RPS15;RPS14;RPS27;RPL10;RPS19;RPL34;RPLP0;RPL37A;RPL13;RPL38
5	ribosome biogenesis (GO:0042254)	10/192	8.92E-16	7.52E-15	108.8	3769.5	RPS15;RPS14;RPS27;RPL10;RPS19;RPL34;RPLP0;RPL37A;RPL13;RPL38
5	ncRNA processing (GO:0034470)	10/201	1.42E-15	1.12E-14	103.6	3542.1	RPS15;RPS14;RPS27;RPL10;RPS19;RPL34;RPLP0;RPL37A;RPL13;RPL38
5	ribosome assembly (GO:0042255)	7/50	2.98E-14	2.20E-13	249.7	7775.7	RPS15;RPS14;RPS27;RPL10;RPS19;RPLP0;RPSA
5	ribonucleoprotein complex assembly (GO:0022618)	8/136	4.36E-13	3.03E-12	103.4	2942.7	RPS15;RPS14;RPS27;RPL10;RPS19;RPLP0;RPSA;RPL38
5	ribosomal small subunit assembly (GO:0000028)	5/17	3.57E-12	2.34E-11	554.7	14619.9	RPS15;RPS14;RPS27;RPS19;RPSA
5	ribosomal small subunit biogenesis (GO:0042274)	5/40	3.74E-10	2.33E-09	190.0	4123.1	RPS15;RPS14;RPS27;RPS19;RPSA
5	maturation of SSU-rRNA from tricistronic rRNA transcript (SSU-rRNA, 5.8S rRNA, LSU-rRNA) (GO:0000462)	3/30	3.41E-06	2.01E-05	130.4	1641.6	RPS14;RPS19;RPS21
5	ribonucleoprotein complex biogenesis (GO:0022613)	3/60	2.82E-05	1.59E-04	61.7	646.1	RPS15;RPS19;RPL38
5	ribosomal large subunit assembly (GO:0000027)	2/25	2.81E-04	1.51E-03	96.4	788.3	RPL10;RPLP0
5	maturation of SSU-rRNA (GO:0030490)	2/35	5.54E-04	2.84E-03	67.2	503.6	RPS14;RPS19
5	erythrocyte differentiation (GO:0030218)	2/46	9.58E-04	4.71E-03	50.3	349.9	RPS14;RPS19
5	myeloid cell differentiation (GO:0030099)	2/52	1.22E-03	5.77E-03	44.3	297.0	RPS14;RPS19
5	ribosomal large subunit biogenesis (GO:0042273)	2/57	1.47E-03	6.66E-03	40.3	262.6	RPL10;RPLP0
5	regulation of respiratory burst involved in inflammatory response (GO:0060264)	1/5	4.99E-03	2.10E-02	262.8	1393.1	RPS19
5	cellular response to erythropoietin (GO:0036018)	1/5	4.99E-03	2.10E-02	262.8	1393.1	MT2A
5	macromolecule biosynthetic process (GO:0009059)	1/6	5.99E-03	2.44E-02	210.3	1076.2	RPS23
5	aldehyde biosynthetic process (GO:0046184)	1/7	6.98E-03	2.50E-02	175.2	869.9	TPI1
5	ribosomal small subunit export from nucleus (GO:0000056)	1/7	6.98E-03	2.50E-02	175.2	869.9	RPS15
5	positive regulation of respiratory burst (GO:0060267)	1/7	6.98E-03	2.50E-02	175.2	869.9	RPS19
5	glyceraldehyde-3-phosphate metabolic process [illegible]	1/7	6.98E-03	2.50E-02	175.2	869.9	TPI1
5	endonucleolytic cleavage in ITS1 to separate SSU-rRNA from 5.8S rRNA and LSU-rRNA from tricistronic rRNA transcript (SSU-rRNA, 5.8S rRNA, LSU-rRNA) (GO:0000447)	1/8	7.97E-03	2.77E-02	150.2	725.6	RPS21
5	ketone biosynthetic process (GO:0042181)	1/9	8.97E-03	3.02E-02	131.4	619.4	TPI1
5	rRNA 3'-end processing (GO:0031125)	1/10	9.96E-03	3.26E-02	116.8	538.3	RPS21
5	glycerol metabolic process (GO:0006071)	1/11	1.09E-02	3.49E-02	105.1	474.5	TPI1
5	ribosomal subunit export from nucleus (GO:0000054)	1/13	1.29E-02	3.91E-02	87.6	380.8	RPS15
5	endonucleolytic cleavage of tricistronic rRNA transcript (SSU-rRNA, 5.8S rRNA, LSU-rRNA) (GO:0000479)	1/13	1.29E-02	3.91E-02	87.6	380.8	RPS21
5	cellular copper ion homeostasis (GO:0006878)	1/14	1.39E-02	4.10E-02	80.8	345.6	MT2A

5	Arp2/3 complex-mediated actin nucleation (GO:0034314)	1/15	1.49E-02	4.29E-02	75.1	315.7	ARPC5L
5	negative regulation of immune effector process [illegible]	1/16	1.59E-02	4.46E-02	70.1	290.2	RPS19
5	negative regulation of viral entry into host cell [illegible]	1/17	1.69E-02	4.52E-02	65.7	268.1	LY6E
5	copper ion homeostasis (GO:0055070)	1/17	1.69E-02	4.52E-02	65.7	268.1	MT2A
5	cellular response to zinc ion (GO:0071294)	1/18	1.79E-02	4.68E-02	61.8	248.8	MT2A
5	cellular response to copper ion (GO:0071280)	1/19	1.88E-02	4.83E-02	58.4	231.8	MT2A
5	negative regulation of viral life cycle (GO:1903901)	1/20	1.98E-02	4.98E-02	55.3	216.8	LY6E
6	cardiolipin biosynthetic process (GO:0032049)	1/7	7.00E-04	2.70E-03	3332.0	24205.7	CRLS1
6	glycerolipid biosynthetic process (GO:0045017)	1/9	9.00E-04	2.70E-03	2498.8	17524.6	CRLS1
6	phosphatidylglycerol biosynthetic process (GO:0006655)	1/9	9.00E-04	2.70E-03	2498.8	17524.6	CRLS1
6	cardiolipin metabolic process (GO:0032048)	1/14	1.40E-03	3.15E-03	1537.3	10102.6	CRLS1
6	phosphatidylglycerol acyl-chain remodeling (GO:0036148)	1/18	1.80E-03	3.24E-03	1175.4	7428.7	CRLS1
6	phosphatidylglycerol metabolic process (GO:0046471)	1/25	2.50E-03	3.75E-03	832.3	4986.9	CRLS1
6	phospholipid biosynthetic process (GO:0008654)	1/37	3.70E-03	4.75E-03	554.5	3105.4	CRLS1
6	glycerophospholipid metabolic process (GO:0006650)	1/80	7.98E-03	8.98E-03	252.1	1217.9	CRLS1
6	glycerophospholipid biosynthetic process (GO:0046474)	1/177	1.76E-02	1.76E-02	112.6	454.8	CRLS1
7	negative regulation of cardiac muscle cell apoptotic process (GO:0010667)	1/6	2.40E-03	3.18E-02	571.1	3445.3	PPP1R10
7	arginine catabolic process (GO:0006527)	1/7	2.80E-03	3.18E-02	475.9	2797.7	OAT
7	regulation of myosin-light-chain-phosphatase activity [illegible]	1/7	2.80E-03	3.18E-02	475.9	2797.7	PPP1R12A
7	negative regulation of striated muscle cell apoptotic process (GO:0010664)	1/8	3.20E-03	3.18E-02	407.9	2343.5	PPP1R10
7	regulation of endothelial cell development (GO:1901550)	1/9	3.59E-03	3.18E-02	356.9	2008.5	PPP1R12A
7	RNA polymerase II preinitiation complex assembly [illegible]	1/10	3.99E-03	3.18E-02	317.2	1751.9	GTF2A2
7	regulation of cardiac muscle cell apoptotic process [illegible]	1/11	4.39E-03	3.18E-02	285.5	1549.4	PPP1R10
7	cytoplasmic sequestering of protein (GO:0051220)	1/13	5.19E-03	3.18E-02	237.9	1251.4	YWHAB
7	transcription preinitiation complex assembly (GO:0070897)	1/13	5.19E-03	3.18E-02	237.9	1251.4	GTF2A2
7	positive regulation of chromosome organization [illegible]	1/15	5.99E-03	3.18E-02	203.9	1043.4	PPP1R10
7	regulation of establishment of endothelial barrier [illegible]	1/17	6.78E-03	3.18E-02	178.4	890.7	PPP1R12A
7	positive regulation of phosphoprotein phosphatase activity (GO:0032516)	1/18	7.18E-03	3.18E-02	167.9	828.7	PPP1R12A
7	negative regulation of dephosphorylation (GO:0035305)	1/20	7.97E-03	3.18E-02	150.2	725.6	YWHAB
7	cobalamin metabolic process (GO:0009235)	1/21	8.37E-03	3.18E-02	142.7	682.3	MMADHC
7	negative regulation of protein dephosphorylation [illegible]	1/22	8.77E-03	3.18E-02	135.9	643.5	YWHAB

7	tetrapyrrole metabolic process (GO:0033013)	1/22	8.77E-03	3.18E-02	135.9	643.5	MMADHC
7	glutamate metabolic process (GO:0006536)	1/22	8.77E-03	3.18E-02	135.9	643.5	OAT
7	negative regulation of G protein-coupled receptor signaling pathway (GO:0045744)	1/23	9.16E-03	3.18E-02	129.7	608.5	YWHAB
7	regulation of telomere maintenance (GO:0032204)	1/26	1.04E-02	3.18E-02	114.1	521.5	PPP1R10
7	hippo signaling (GO:0035329)	1/26	1.04E-02	3.18E-02	114.1	521.5	YWHAB
7	regulation of protein insertion into mitochondrial membrane involved in apoptotic signaling pathway	1/26	1.04E-02	3.18E-02	114.1	521.5	YWHAB
7	positive regulation of protein insertion into mitochondrial membrane involved in apoptotic signaling pathway	1/26	1.04E-02	3.18E-02	114.1	521.5	YWHAB
7	negative regulation of catalytic activity (GO:0043086)	1/27	1.08E-02	3.18E-02	109.7	497.3	PPP1R12A
7	positive regulation of mitotic cell cycle (GO:0045931)	1/27	1.08E-02	3.18E-02	109.7	497.3	PPP1R10
7	positive regulation of telomere maintenance (GO:0032206)	1/32	1.27E-02	3.62E-02	92.0	401.4	PPP1R10
7	positive regulation of mitochondrial outer membrane permeabilization involved in apoptotic signaling pathway (GO:1901030)	1/34	1.35E-02	3.69E-02	86.4	371.8	YWHAB
7	glutamine family amino acid metabolic process [illegible]	1/37	1.47E-02	3.83E-02	79.2	334.1	OAT
7	regulation of phosphoprotein phosphatase activity [illegible]	1/38	1.51E-02	3.83E-02	77.0	323.1	PPP1R12A
7	positive regulation of catalytic activity (GO:0043085)	1/40	1.59E-02	3.85E-02	73.1	302.7	YWHAB
7	regulation of protein dephosphorylation (GO:0035304)	1/41	1.63E-02	3.85E-02	71.3	293.4	YWHAB
7	alpha-amino acid metabolic process (GO:1901605)	1/46	1.83E-02	4.18E-02	63.3	253.5	OAT
7	positive regulation of establishment of protein localization to mitochondrion (GO:1903749)	1/56	2.22E-02	4.92E-02	51.8	197.2	YWHAB
8	TORC2 signaling (GO:0038203)	1/6	3.00E-04	1.80E-03	19994.0	162186.5	PRR5
8	TOR signaling (GO:0031929)	1/26	1.30E-03	3.10E-03	19974.0	132735.5	PRR5
8	activation of protein kinase B activity (GO:0032148)	1/31	1.55E-03	3.10E-03	19969.0	129189.9	PRR5
8	activation of protein kinase activity (GO:0032147)	1/114	5.70E-03	8.55E-03	19886.0	102757.0	PRR5
8	regulation of signal transduction by p53 class mediator [illegible]	1/156	7.80E-03	9.36E-03	19844.0	96315.7	PRR5
8	regulation of intracellular signal transduction [illegible]	1/437	2.18E-02	2.18E-02	19563.0	74800.3	PRR5
